# Integrative analysis of transcriptome, DNA methylome, and chromatin accessibility reveals candidate therapeutic targets in hypertrophic cardiomyopathy

**DOI:** 10.1093/procel/pwae032

**Published:** 2024-05-23

**Authors:** Junpeng Gao, Mengya Liu, Minjie Lu, Yuxuan Zheng, Yan Wang, Jingwei Yang, Xiaohui Xue, Yun Liu, Fuchou Tang, Shuiyun Wang, Lei Song, Lu Wen, Jizheng Wang

**Affiliations:** Biomedical Pioneering Innovation Center, School of Life Sciences, Peking University, Beijing 100871, China; Emergency Center, Zhongnan Hospital of Wuhan University, Wuhan 430071, China; Beijing Advanced Innovation Center for Genomics (ICG), Ministry of Education Key Laboratory of Cell Proliferation and Differentiation, Beijing 100871, China; Biomedical Pioneering Innovation Center, School of Life Sciences, Peking University, Beijing 100871, China; Beijing Advanced Innovation Center for Genomics (ICG), Ministry of Education Key Laboratory of Cell Proliferation and Differentiation, Beijing 100871, China; State Key Laboratory of Cardiovascular Disease, Fuwai Hospital, National Center for Cardiovascular Disease, Chinese Academy of Medical Science and Peking Union Medical College, Beijing 100037, China; Biomedical Pioneering Innovation Center, School of Life Sciences, Peking University, Beijing 100871, China; Beijing Advanced Innovation Center for Genomics (ICG), Ministry of Education Key Laboratory of Cell Proliferation and Differentiation, Beijing 100871, China; Peking-Tsinghua Center for Life Sciences, Academy for Advanced Interdisciplinary Studies, Peking University, Beijing 100871, China; Biomedical Pioneering Innovation Center, School of Life Sciences, Peking University, Beijing 100871, China; Beijing Advanced Innovation Center for Genomics (ICG), Ministry of Education Key Laboratory of Cell Proliferation and Differentiation, Beijing 100871, China; Biomedical Pioneering Innovation Center, School of Life Sciences, Peking University, Beijing 100871, China; Beijing Advanced Innovation Center for Genomics (ICG), Ministry of Education Key Laboratory of Cell Proliferation and Differentiation, Beijing 100871, China; Biomedical Pioneering Innovation Center, School of Life Sciences, Peking University, Beijing 100871, China; Beijing Advanced Innovation Center for Genomics (ICG), Ministry of Education Key Laboratory of Cell Proliferation and Differentiation, Beijing 100871, China; Biomedical Pioneering Innovation Center, School of Life Sciences, Peking University, Beijing 100871, China; Beijing Advanced Innovation Center for Genomics (ICG), Ministry of Education Key Laboratory of Cell Proliferation and Differentiation, Beijing 100871, China; Biomedical Pioneering Innovation Center, School of Life Sciences, Peking University, Beijing 100871, China; Beijing Advanced Innovation Center for Genomics (ICG), Ministry of Education Key Laboratory of Cell Proliferation and Differentiation, Beijing 100871, China; Peking-Tsinghua Center for Life Sciences, Academy for Advanced Interdisciplinary Studies, Peking University, Beijing 100871, China; Department of Cardiovascular Surgery, Fuwai Hospital, National Center for Cardiovascular Diseases, Chinese Academy of Medical Sciences and Peking Union Medical College, Beijing 100037, China; State Key Laboratory of Cardiovascular Disease, Fuwai Hospital, National Center for Cardiovascular Disease, Chinese Academy of Medical Science and Peking Union Medical College, Beijing 100037, China; Cardiomyopathy Ward, Fuwai Hospital, National Center for Cardiovascular Diseases, Chinese Academy of Medical Sciences and Peking Union Medical College, Beijing 100037, China; National Clinical Research Center for Cardiovascular Diseases, Fuwai Hospital, National Center for Cardiovascular Diseases, Chinese Academy of Medical Sciences and Peking Union Medical College, Beijing 100037, China; Biomedical Pioneering Innovation Center, School of Life Sciences, Peking University, Beijing 100871, China; Beijing Advanced Innovation Center for Genomics (ICG), Ministry of Education Key Laboratory of Cell Proliferation and Differentiation, Beijing 100871, China; State Key Laboratory of Cardiovascular Disease, Fuwai Hospital, National Center for Cardiovascular Disease, Chinese Academy of Medical Science and Peking Union Medical College, Beijing 100037, China

**Keywords:** hypertrophic cardiomyopathy, multi-omics, chromatin accessibility, DNA methylation, fetal gene reprogramming, therapy

## Abstract

Hypertrophic cardiomyopathy (HCM) is the most common inherited heart disease and is characterized by primary left ventricular hypertrophy usually caused by mutations in sarcomere genes. The mechanism underlying cardiac remodeling in HCM remains incompletely understood. An investigation of HCM through integrative analysis at multi-omics levels will be helpful for treating HCM. DNA methylation and chromatin accessibility, as well as gene expression, were assessed by nucleosome occupancy and methylome sequencing (NOMe-seq) and RNA-seq, respectively, using the cardiac tissues of HCM patients. Compared with those of the controls, the transcriptome, DNA methylome, and chromatin accessibility of the HCM myocardium showed multifaceted differences. At the transcriptome level, HCM hearts returned to the fetal gene program through decreased sarcomeric and metabolic gene expression and increased extracellular matrix gene expression. In the DNA methylome, hypermethylated and hypomethylated differentially methylated regions were identified in HCM. At the chromatin accessibility level, HCM hearts showed changes in different genome elements. Several transcription factors, including SP1 and EGR1, exhibited a fetal-like pattern of binding motifs in nucleosome-depleted regions in HCM. In particular, the inhibition of SP1 or EGR1 in an HCM mouse model harboring sarcomere mutations markedly alleviated the HCM phenotype of the mutant mice and reversed fetal gene reprogramming. Overall, this study not only provides a high-precision multi-omics map of HCM heart tissue but also sheds light on the therapeutic strategy by intervening in the fetal gene reprogramming in HCM.

## Introduction

Hypertrophic cardiomyopathy (HCM) is one of the most common inherited cardiovascular diseases and is characterized by primary left ventricular hypertrophy (Maron, 2002, 2004, 2014). Its prevalence is estimated to be as high as 1 in 200 people in the general population (Maron et al., 1995; Semsarian et al., 2015). HCM is an important cause of cardiovascular morbidity and mortality, especially sudden cardiac death (SCD) and heart failure (Maron, 2010; Maron and Maron, 2013). Genetic studies across more than three decades have revealed that mutations in genes encoding cardiac sarcomere proteins are the main causes, accounting for approximately half of the disease (Marian and Braunwald, 2017; Walsh et al., 2017). Although the causality of sarcomere mutations has been well established, the molecular processes at different regulatory levels that mediate the development of this disease, from genetic defects to cardiac hypertrophy, remain largely unknown.

In recent years, omics technologies have been applied to reveal molecular changes in HCM. Bulk RNA-seq studies on cardiac tissues have revealed the transcriptome characteristics of HCM (Gao et al., 2020; Liu et al., 2019; Newman et al., 2017; Ren et al., 2016). Proteomic analysis of myocardial samples from HCM patients has been performed (Coats et al., 2018; Schuldt et al., 2021; Tucholski et al., 2020). Recently, single-cell RNA sequencing was used to analyze heart tissues from HCM patients, which revealed the pathogenesis of HCM at single-cell resolution (Liu et al., 2023; Wehrens et al., 2022). Most relevant studies involve transcriptomic or proteomic analyses. Multi-omics analysis can provide a better understanding of the relationships among different omics. However, only a few studies have analyzed HCM at multi-omics levels, and they have focused mainly on the relationships between proteomes and other omics, such as transcriptomics, metabolomics, and H3K27 acetylation (Garmany et al., 2023; Pei et al., 2021; Previs et al., 2022). A comprehensive multi-omics analysis of HCM including gene expression, DNA methylation, and chromatin accessibility at single-base resolution, is still lacking.

Nucleosome occupancy and methylome sequencing (NOMe-seq) is a single-base resolution sequencing methodology that acquires chromatin accessibility and endogenous DNA methylation information from the same individual DNA molecule in a single workflow (Kelly et al., 2012). Both chromatin accessibility and DNA methylation are pivotal epigenetic layers for regulating gene expression (Jones, 2012; Klemm et al., 2019). NOMe-seq has been applied in resolving the cancer epigenome and epigenomic reprogramming of fetal germ cells (Guo et al., 2017; Lay et al., 2015; Taberlay et al., 2014). In 2021, we reported a multi-omics map of healthy human and mouse hearts using NOMe-seq and RNA-seq, serving as a reference for developing novel therapeutic strategies compared with cardiac tissue samples of cardiovascular diseases (CVDs) (Gao et al., 2021). In this study, we applied NOMe-seq and RNA-seq to cardiac tissues from HCM patients to map integrated omics, including transcriptome, DNA methylome, and chromatin accessibility, at single-base resolution. Potential pivotal transcription factors (TFs) during the chromatin accessibility remodeling were identified in HCM. We also constructed an HCM mouse model (*Myh6*^*R454C*/+^*Tnnt2*^*R127W*/+^) using CRISPR/Cas9-mediated targeted integration. Preliminary experimental evidence showed that plicamycin and ML264 significantly alleviated hypertrophy symptoms by targeting and repressing the transcription factors SP1 and EGR1, respectively, in HCM mouse model.

## Results

### Comparative transcriptomic analysis of HCM patients and healthy donors

**Figure 1.**
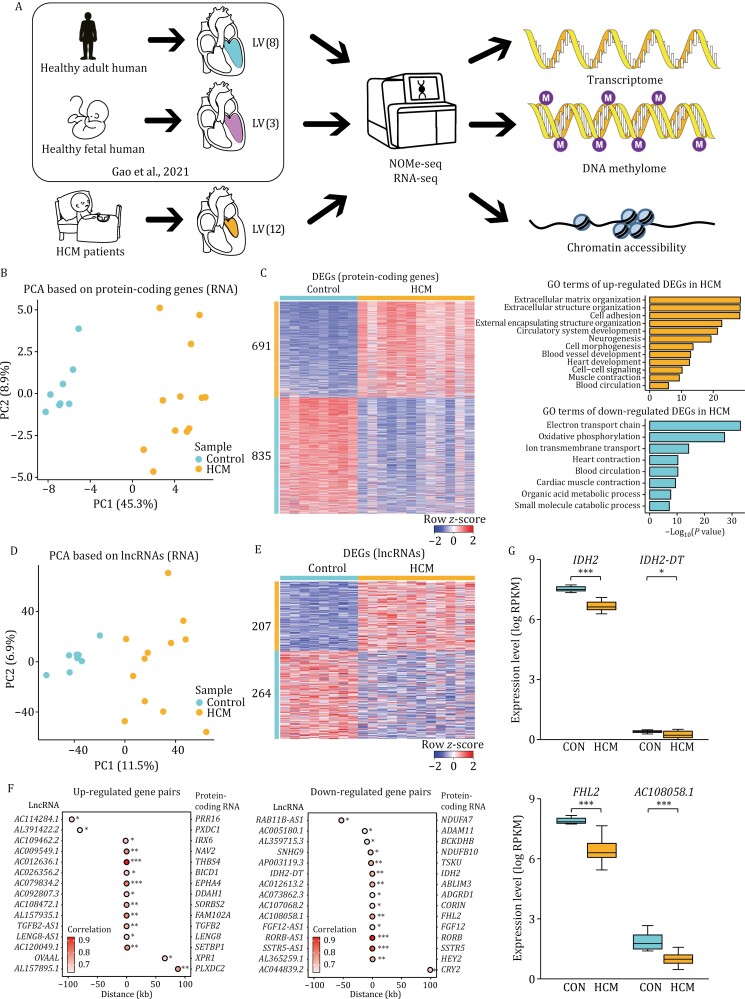
Transcriptomics profiles of HCM patients and healthy adult donors. (A) Schematic diagram of experimental design. The number of samples used for each group was indicated in brackets. (B) PCA plot showing the transcriptome pattern of protein-coding genes in HCM patients and healthy donors. PC1 and PC2 had variance values of 45.3% and 8.9%, respectively. (C) Heatmap showing row *z*-score scaled gene expression levels of DEGs (protein-coding genes) between HCM patients and healthy donors, and related GO terms were displayed on the right. (D) PCA plot showing the transcriptome pattern of lncRNAs in HCM patients and healthy donors. PC1 and PC2 had variance values of 11.5% and 6.9%, respectively. (E) Heatmap showing row *z*-score scaled gene expression levels of DEGs (lncRNAs) between HCM patients and healthy donors. (F) Dot plots showing gene pairs between lncRNAs (left) and protein-coding genes (right) in analysis of *cis*-regulatory relationships. The x-axis shows the linear distance between the lncRNAs and the protein-coding genes. The colors of the dots represented the Pearson correlation coefficient, and stars represented the correlation significance. Statistical significance was performed by Pearson correlation test. (G) Boxplots showing the expression levels of representative gene pairs with *cis*-regulatory relationships between HCM patients and controls. Statistical significance was performed by two-tailed Student’s *t* test. *P value ≤ 0.05, **P value ≤ 0.01, ***P value ≤ 0.001.

To explore the pathological mechanism of HCM, myocardium obtained from HCM patients (*n* = 12) was collected for multi-omics analysis (Table S1). We performed NOMe-seq to profile epigenomics, including DNA methylome and chromatin accessibility, and performed ribosomal RNA-depleted RNA-seq to profile the transcriptome (Fig. 1A). The data of left ventricle tissues from normal adult (*n* = 8) and fetal (*n* = 3) hearts, which were generated from the same NOMe-seq and RNA-seq protocols and reported in our previous study, were used as controls for comparison (Gao et al., 2021). NOMe-seq and RNA-seq generated an average of 38.61 Gb and 10.26 Gb of raw data per library, respectively, and 2.01 Tb of sequencing data were generated in total (Table S2).

First, we investigated transcriptome changes in HCM. Principal component analysis (PCA) based on protein-coding genes clearly distinguished the HCM patients from the adult controls (Fig. 1B). We identified differentially expressed genes (DEGs) between the two groups (Table S3; Fig. S1). A total of 691 DEGs, which were enriched in gene ontology (GO) terms including extracellular matrix organization, extracellular structure organization, cell adhesion, and circulatory system development, were up-regulated in the HCM patients. A total of 835 DEGs, which were enriched in the GO terms including electron transport chain, oxidative phosphorylation, heart contraction, and metabolic processes, were down-regulated in HCM patients (Fig. 1C). The up-regulated DEGs mainly reflected activation and excessive accumulation of extracellular matrix proteins in the HCM myocardium (Brower et al., 2006; Lombardi et al., 2003). The down-regulated DEGs were primarily involved in perturbed metabolic signaling, which illustrates that HCM is a disease with altered cardiac energetics.

Long noncoding RNAs (lncRNAs) are also important regulators of cardiac development and homeostasis (Boon et al., 2016; Lorenzen and Thum, 2016; Uchida and Dimmeler, 2015). PCA using only lncRNAs also successfully separated HCM patients from adult controls (Fig. 1D). In total, 207 up-regulated differentially expressed lncRNAs and 264 down-regulated differentially expressed lncRNAs in HCM were identified (Table S3; Fig. 1E). We then examined the correlations between the expression of protein-coding genes and lncRNAs to identify potential *cis*-regulation, and found 15 up-regulated gene pairs and 15 down-regulated gene pairs (Fig. 1F). Among these gene pairs, IDH2 has been shown to play a role in preventing oxidative stress in cardiac hypertrophy in mice (Ku et al., 2015) and FHL2 has been shown to repress pathological cardiac remodeling (Fig. 1G) (Hojayev et al., 2012).

### Genome-wide DNA methylation analysis of HCM versus healthy conditions

**Figure 2.**
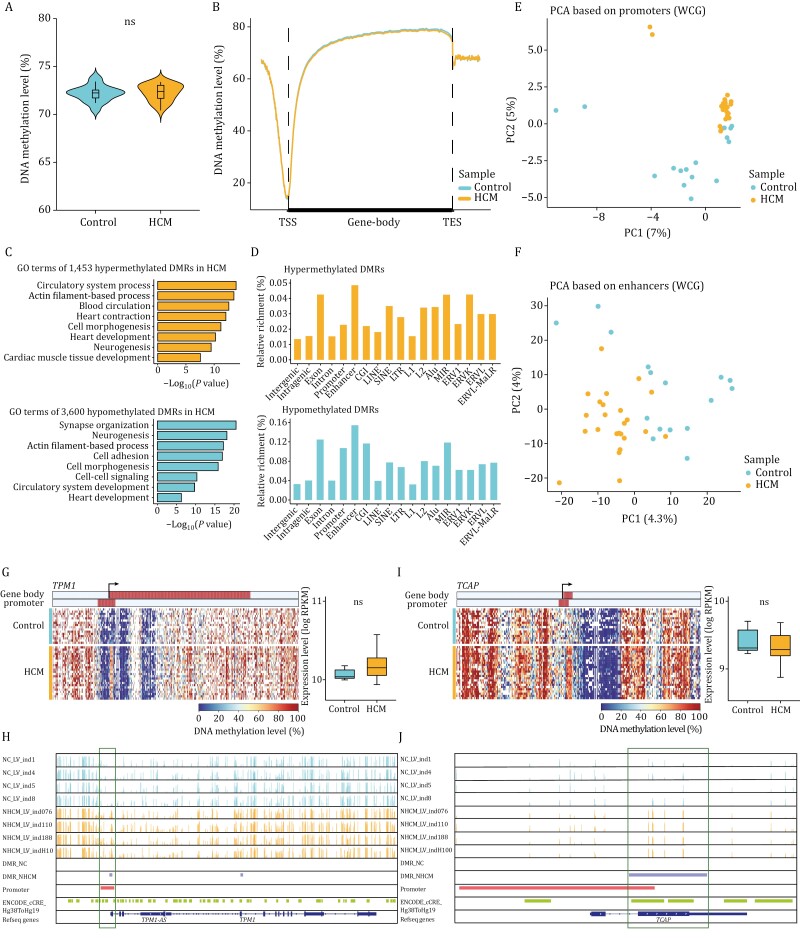
Endogenous DNA methylation analysis of HCM versus healthy conditions. (A) Violin plot showing the genome-wide endogenous DNA methylation levels of HCM patients and healthy donors. ns: *P* value > 0.05. (B) Line plot showing the average endogenous DNA methylation levels in the gene body and 2 kb upstream and downstream regions of gene body in HCM patients and healthy controls. (C) GO terms of genes in which DMRs were located. Top, GO terms of hypermethylated DMRs in the HCM patients; bottom, GO terms of hypomethylated DMRs in the HCM patients. (D) Bar plots showing the relative enrichment of hypermethylated and hypomethylated DMRs located in different genome elements. (E) PCA plot showing the endogenous DNA methylation pattern of promoters in HCM patients and healthy controls. PC1 and PC2 had variance values of 7% and 5%, respectively. (F) PCA plot showing the endogenous DNA methylation pattern of enhancers in HCM patients and healthy controls. PC1 and PC2 had variance values of 4.3% and 4%, respectively. (G) Heatmap showing endogenous DNA methylation levels in the gene body ± 10 kb regions of *TPM1* in all HCM patients and controls. The gene expression levels of *TPM1* were showed in the boxplot on the right, and the gene expression levels were quantified by log_2_(RPKM + 1). The color bars in the heatmap represented the regions of gene body and promoter (from 1 kb upstream of the TSS to 0.5 kb downstream of the TSS), and the direction of the arrow indicated the direction of transcription. ns: *P* value > 0.05. (H) IGV image showing the endogenous DNA methylation levels of *TPM1* and adjacent regions in four HCM patients and four controls. Each vertical line represented a site, and the height of the vertical line represented the endogenous DNA methylation level. DMR_NC and DMR_NHCM represented the hypomethylated DMRs and hypermethylated DMRs identified in HCM patients, respectively. ENCODE_cCRE_Hg38ToHg19 showed the position of the enhancers. (I) Heatmap showing endogenous DNA methylation levels in the gene body ± 10 kb regions (left) and boxplot showing the gene expression levels (right) of *TCAP* in all HCM patients and controls. ns: *P* value > 0.05. (J) IGV image showing the endogenous DNA methylation levels of *TCAP* and adjacent regions in 4 HCM patients and 4 controls. Statistical significance was performed by two-tailed Student’s *t* test.

Next, we investigated DNA methylation changes in the HCM myocardium. Two technical replicates of one sample were highly reproducible (Fig. S2A). The total DNA methylation levels were similar between HCM patients and controls (median 72.40% in HCM patients vs. 72.24% in controls, Fig. 2A). The average DNA methylation level distribution around the gene body and flanking regions between HCM patients and controls almost overlapped (Fig. 2B).

PCA suggested that the DNA methylomes of HCM patients and controls were different (Fig. S2B). We then identified differentially methylated regions (DMRs) between HCM patients and controls using a strict criterion of 20% differences in the methylation level. In total, 1,453 hypermethylated DMRs were identified in HCM patients, and genes associated with these DMRs were enriched in actin filament-based process, blood circulation and heart contraction. A total of 3,600 DMRs were hypomethylated in HCM patients, and the associated genes were enriched in synapse organization, cell adhesion, and cell-cell signaling (Fig. 2C; Table S4). Both the hypermethylated and hypomethylated DMRs were enriched in exons, promoters and enhancer regions (Figs. 2D and S2C).

The general DNA methylation levels at various genome elements, including exons, promoters, and enhancers, were similar between HCM patients and controls (Fig. S2D). PCA based on DNA methylation at promoters, enhancers, gene bodies and CpG islands (CGIs) could distinguish HCM patients from adult controls (Figs. 2E, 2F, S2E and S2F). The methylation levels in the promoters of *TPM1* increased significantly in HCM patients (Fig. 2G and 2H). TPM1 is a sarcomeric protein, and mutations in *TPM1* have been linked to HCM and congenital heart defects (England et al., 2017; Nunez et al., 2013; Sewanan et al., 2021). In the gene-body region, *TCAP* showed significantly higher methylation levels in HCM patients (Fig. 2I and 2J). TCAP is also a sarcomeric protein whose mutations have been found in patients with HCM and dilated cardiomyopathy (DCM) (Hayashi et al., 2004; Hershberger et al., 2008). In addition, *ACTA1* also showed higher methylation levels in gene-body region of HCM patients (Fig. S2G and S2H); *ACTA1* has been reported to be associated with cardiomyopathy (Lim et al., 2001; Tsybouleva et al., 2004).

### Changes of chromatin accessibility in the HCM myocardium

**Figure 3.**
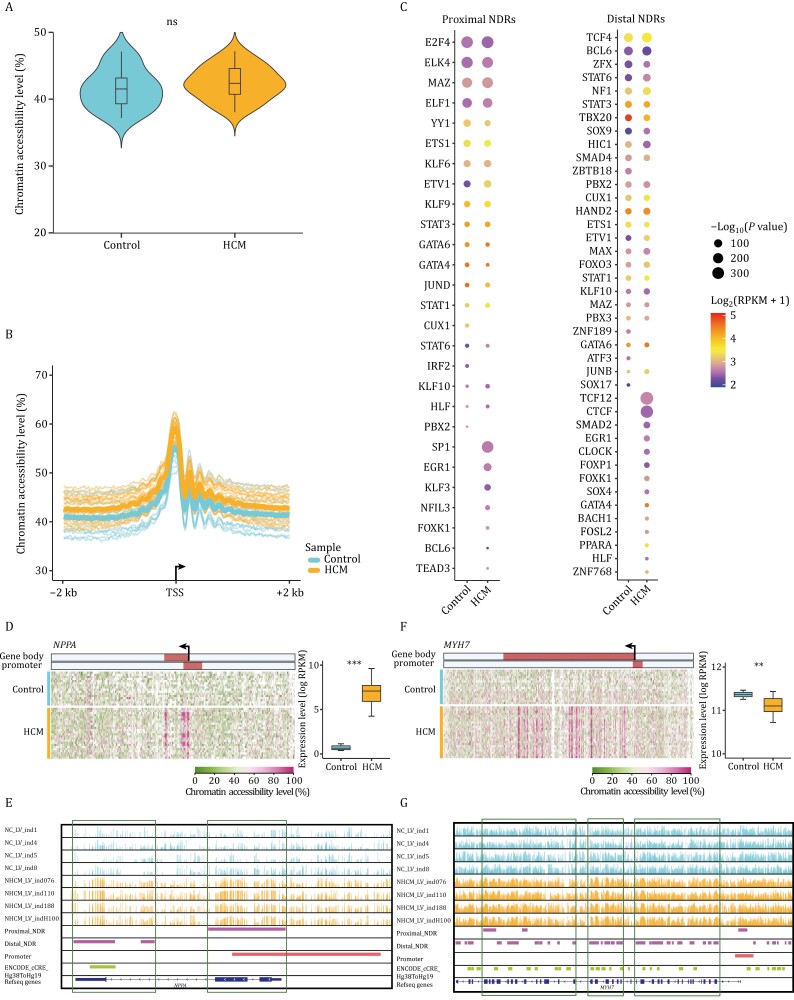
Chromatin accessibility profiles of HCM and healthy adult controls. (A) Violin plot showing the total accessibility levels of whole-genome in HCM patients and healthy adult controls. ns: *P* value > 0.05. (B) Line plot showing the average chromatin accessibility levels in the 2 kb upstream and downstream regions of TSS in HCM patients and controls. (C) Motif enrichment analysis of proximal (left) and distal (right) NDRs in HCM patients and healthy adult controls. The sizes of dots indicated the *P* values (*P* value ≤ 10^−10^) and the colors of dots represented average expression levels of the corresponding transcription factors. (D) Heatmap showing chromatin accessibility levels in the gene body ± 10 kb regions of *NPPA* in all HCM patients and controls. The gene expression levels of *NPPA* were showed in the boxplot on the right, and the gene expression levels were quantified by log_2_(RPKM + 1). The color bars in the heatmap represented the regions of gene body and promoter (from 1 kb upstream of the TSS to 0.5 kb downstream of the TSS), and the direction of the arrow indicated the direction of transcription. ***P value ≤ 0.001. (E) IGV image showing the chromatin accessibility levels of *NPPA* and adjacent regions in four HCM patients and four controls. Each vertical line represented a site, and the height of the vertical line represented the chromatin accessibility level. Proximal_NDR and Distal_NDR represented the proximal NDRs and distal NDRs identified in HCM patients and controls, respectively. ENCODE_cCRE_Hg38ToHg19 showed the position of the enhancers. (F) Heatmap showing chromatin accessibility levels in the gene body ± 10 kb regions (left) and boxplot showing the gene expression levels (right) of *MYH7* in all HCM patients and controls. **P value ≤ 0.01. (G) IGV image showing the chromatin accessibility levels of *MYH7* and adjacent regions in four HCM patients and four controls. Statistical significance was performed by two-tailed Student’s *t* test.

To map the genome-wide chromatin accessibility landscape of HCM, we calculated chromatin accessibility by the cytosine methylation level in the GCH (GCA/GCT/GCC) context. The chromatin accessibility at the whole-genome level, and the distributions of chromatin accessibility around the transcription start site (TSS) were similar between HCM patients and controls (Fig. 3A and 3B). The distributions of chromatin accessibility levels between two technical replicates were highly replicable (Fig. S3A). Nucleosome-depleted regions (NDRs) usually harbor binding sites of TFs that are relevant to active DNA regulatory elements. In total, we identified 30,087 proximal NDRs and 484,078 distal NDRs by integrating data from HCM patients and healthy adult donors. Compared with those in the controls, the chromatin accessibility levels of proximal NDRs in HCM patients were significantly increased (Fig. S3B). The chromatin accessibility levels of distal NDRs in HCM patients were higher than those in the controls, yet the difference was not significant. PCA showed that the chromatin accessibility of the proximal or distal NDRs distinguished the HCM patients from the controls (Fig. S3C and S3D). We identified TF binding motifs enriched in the proximal and distal NDRs of HCM patients and healthy adult controls. HCM patients and healthy adult controls shared binding motifs, including STATs (STAT1, STAT3, and STAT6), GATA6, and MAZ (Fig. 3C). In our previous study, the binding motifs of these TFs were also enriched in four chambers of human and mouse hearts, indicating the conserved functions of these TFs (Gao et al., 2021).

Dynamic changes in chromatin accessibility in HCM patients were also assessed for different genome elements. PCA clearly separated the HCM patients from the controls based on the chromatin accessibility of the promoter, gene body, and enhancer (Fig. S3E–G). *NPPA* is a typical HCM marker gene and several key TFs regulate the proximal *NPPA* promoter (Chien et al., 1991; De Bold et al., 1981; Goetze et al., 2020; Houweling et al., 2005). We found that the chromatin accessibility level of the *NPPA* promoter in HCM patients was markedly increased (Fig. 3D and 3E). *MYH7* encodes the β-myosin heavy chain and is one of the most common genes mutated in HCM (Geisterfer-Lowrance et al., 1990; Marian and Braunwald, 2017). The gene body of *MYH7* in HCM patients showed higher chromatin accessibility levels than that in the controls (Fig. 3F and 3G). The chromatin accessibility patterns of these key HCM genes indicated that changes in chromatin accessibility play an important role in the pathological remodeling of HCM.

### Correlations among the transcriptome, DNA methylome, and chromatin accessibility in HCM

Then, we integrated three omics data to explore the correlations among them in HCM patients. The results showed that the DNA methylation level around the TSS was negatively correlated with gene expression in HCM patients (Fig. S4A). In contrast, the chromatin accessibility level around the TSS was positively correlated with gene expression (Fig. S4B). The promoter region showed similar correlation patterns (Fig. S4C). On the gene body, both the DNA methylation level and chromatin accessibility displayed bell-shaped relationships with gene expression (Fig. S4D and S4E).

### The HCM heart demonstrates fetal gene reprogramming at both the transcriptome and chromatin accessibility levels

**Figure 4.**
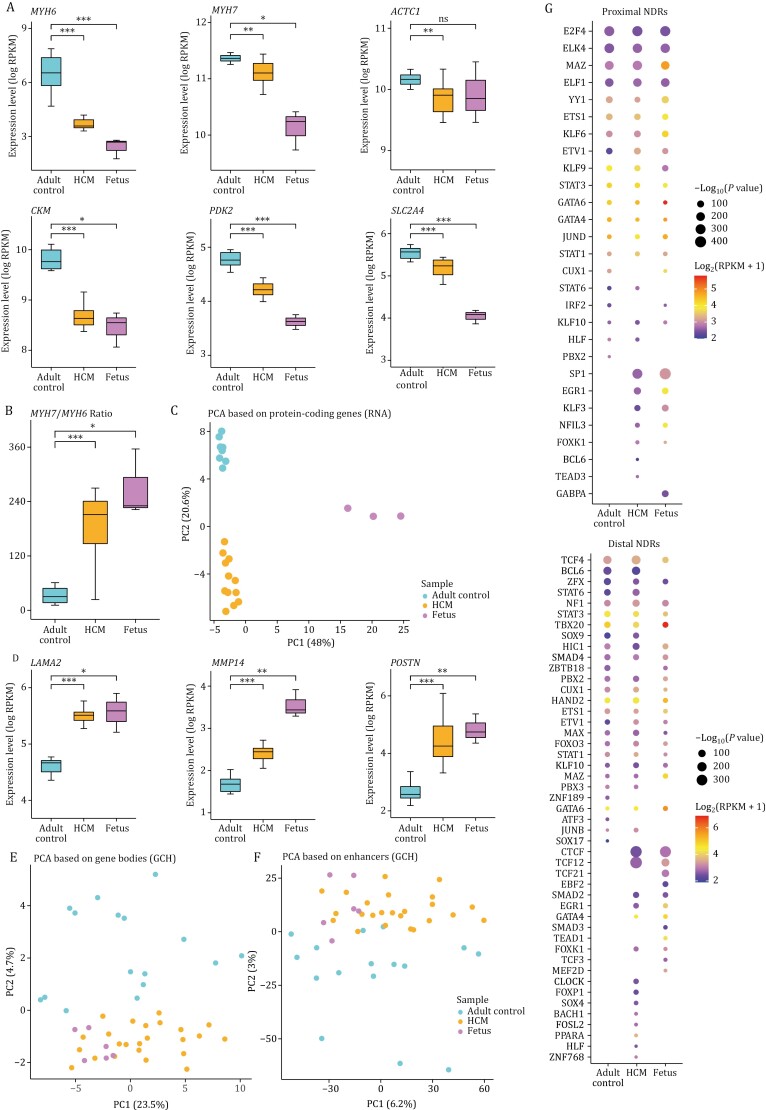
Comparisons of the transcriptome, DNA methylome, and chromatin accessibility in the adult controls, HCM patients, and fetuses. (A) Boxplots showing the expression levels of *MYH6*, *MHY7*, *ACTC1*, *CKM*, *PDK2,* and *SLC2A4* in the adult controls, HCM patients, and fetuses. (B) Boxplot showing the *MYH7/MYH6* ratio in three groups. (C) PCA plot showing the transcriptome pattern of protein-coding genes in the adult controls, HCM patients, and fetuses. PC1 and PC2 had variance values of 48.0% and 20.6%, respectively. (D) Boxplots showing the expression levels of *LAMA2*, *MMP14,* and *POSTN* in the adult controls, HCM patients, and fetuses. (E) PCA plot showing the chromatin accessibility pattern of gene-bodies in the adult controls, HCM patients, and fetuses. PC1 and PC2 had variance values of 23.5% and 4.7%, respectively. (F) PCA plot showing the chromatin accessibility pattern of enhancers in three groups. PC1 and PC2 had variance values of 6.2% and 3.0%, respectively. (G) Motif enrichment analysis of proximal (top) and distal (bottom) NDRs in the adult controls, HCM patients, and fetuses. The sizes of dots indicated the *P* values (*P* value ≤ 10^−10^) of the corresponding transcription factors, and the colors of dots represented average expression levels. Gene expression levels were quantified with log_2_(RPKM + 1). ns: *P* value > 0.05, *P value ≤ 0.05, **P value ≤ 0.01, ***P value ≤ 0.001. Statistical significance was performed by two-tailed Student’s *t* test.

Previous studies have suggested that the adult heart may return to a fetal stage of gene expression profile during pathological remodeling triggered by various stresses, including genetic mutations in contractile proteins (Depre et al., 1998; Mercadier et al., 1981; Rajabi et al., 2007; Razeghi et al., 2001). After integrating our previously published fetal data (Gao et al., 2021), the results showed that key sarcomeric genes, such as *MYH6*, *MYH7,* and *ACTC1*, were expressed at lower levels in both the HCM and fetal myocardium than in the adult controls (Fig. 4A). However, the ratio of *MYH7*/*MYH6* was increased in both HCM patients and fetuses, which indicated MHC-α (encoded by *MYH6*) and MHC-β (encoded by *MYH7*) isoform switched (Fig. 4B). Decreased transcriptional levels of key regulators of energy substrate metabolism, such as *CKM*, *PDK2* and *SLC2A4*, were also detected in both HCM patients and fetuses (Fig. 4A). Then, we explored the fetal gene expression pattern in HCM at the whole-transcriptome level. PCA clearly revealed that the PC2 axis separated the HCM and fetal samples from the adult controls (Fig. 4C). Compared with those in the adult controls, 297 protein-coding genes, which were enriched in GO terms related to an extracellular matrix organization, heart development, and muscle structure development, were co-upregulated in both the fetuses and HCM patients (Fig. S5A and S5B; Table S5). *LAMA2*, *MMP14* and *POSTN* were representative co-upregulated DEGs in both the fetuses and the HCM patients compared with the adult controls (Fig. 4D). These genes have been reported to be involved in extracellular matrix organization and play important roles in HCM (Jaffre et al., 2019; Teekakirikul et al., 2010; Tsoutsman et al., 2013). A total of 524 co-downregulated DEGs in both the fetuses and the HCM patients were enriched in the GO terms electron transport chain and oxidative phosphorylation, among others, which revealed a switch to a fetal state of energy metabolism in HCM (Fig. S5A and S5B; Table S5). Our study showed that HCM patients tended to return to a fetal gene profile by downregulating the expression of sarcomeric and metabolic proteins and upregulating the expression of extracellular matrix genes.

With the multi-omics data, we next explored the reactivation of fetal genes at the epigenomic level. The global DNA methylation levels were similar among the adult controls, the HCM patients, and the fetuses, which indicated a stable DNA methylation pattern in the myocardium (Fig. S5C). Across various genome elements, the PC1 axis was mainly separated into samples based on the developmental stage, and the HCM patients were indistinguishable from the adult controls (Fig. S5D–F), indicating a lack of global fetal reprogramming in HCM at the DNA methylome. Chromatin accessibility at the whole-genome level was also similar among the adult controls, the HCM patients, and the fetuses (Fig. S5G). Nevertheless, using the chromatin accessibility of the gene body or the enhancer, HCM patients were closer to the fetuses, both of which were separated from healthy adult controls (Fig. 4E and 4F). We then identified 31,092 proximal NDRs and 545,960 distal NDRs by integrating data from fetuses, adult controls, and HCM patients (See Methods). The PCA using the chromatin accessibility of the distal NDRs also revealed that the HCM patients were closer to the fetuses than the adult controls, suggesting fetal reprogramming of chromatin accessibility in HCM, which was confirmed by clustering analysis (Fig. S5H and S5I). We analyzed the preferential enrichment patterns of TFs binding motifs in the proximal and distal NDRs of the three groups (Table S6). Several TFs were enriched in both fetuses and HCM patients (e.g., SP1, EGR1, and TCF12). Others were enriched in neither of them but in the healthy adult controls (e.g., PBX2 and SOX17) (Fig. 4G). These results suggested that these TFs might play roles in fetal gene reprogramming in pathological cardiac remodeling and gave cues for new therapeutic targets of HCM.

### Inhibition of the transcription factors SP1 and EGR1 alleviates HCM in mice

To explore whether alterations in chromatin accessibility for TFs are involved in the pathogenesis of HCM, we selected SP1 and EGR1, two zinc finger transcription factors (Cook et al., 1999; Gashler and Sukhatme, 1995), for further experimental analysis. SP1 binding motifs were enriched in the proximal NDRs, and EGR1 binding motifs were enriched in the proximal and distal NDRs of both the HCM and the fetal myocardium, but not in the adult controls (Fig. 4G). The RNA-seq data showed that the expression levels of *SP1* and *EGR1* in HCM patients were higher than those in adult controls, which was significant for *EGR1* but not *SP1* (Fig. S6A and S6B). Real-time quantitative PCR (RT-qPCR) and Western blot showed that, compared with those in the controls, the expression levels of both *SP1* and *EGR1* were significantly higher in the HCM patients at the mRNA and protein levels, respectively (Fig. S6C–E).

**Figure 5.**
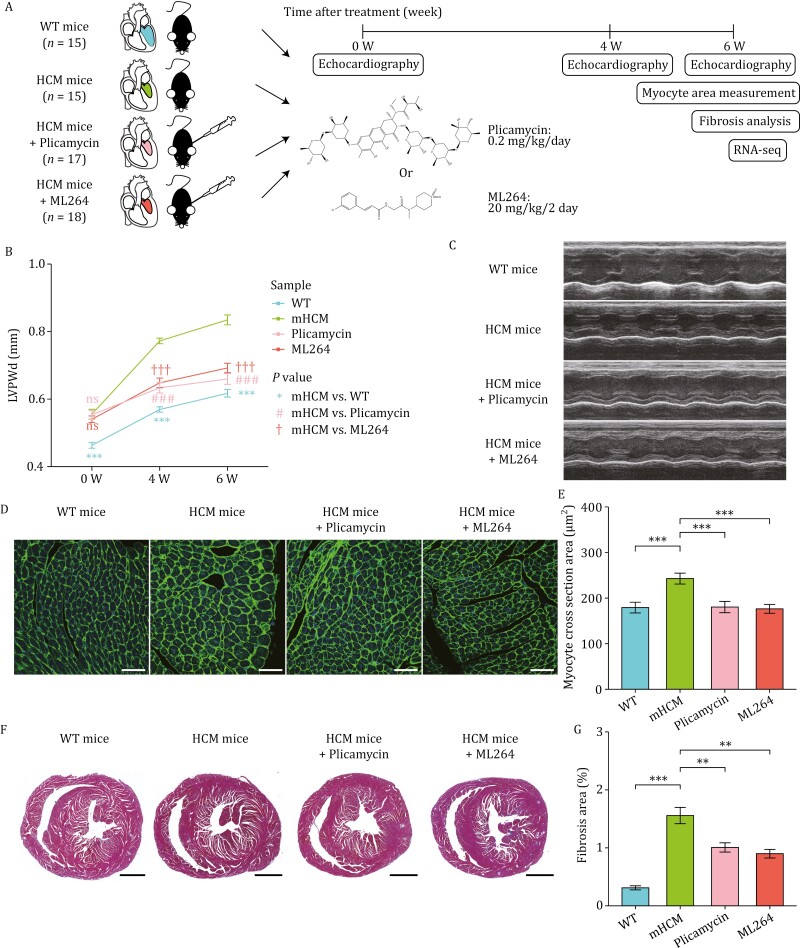
The comparison of physiological and pathological indicators in WT mice, HCM mice, HCM mice treated with plicamycin or ML264. (A) Schematic diagram showing the experimental procedure of plicamycin or ML264 injection into HCM mice. The number of mice in each group and dosage of plicamycin or ML264 were indicated. And some independent experiments were performed for each group at different time points. (B) Line plot showing changes in LVPWd thickness over time by echocardiography for WT mice (blue line) and the indicated HCM mice (green, untreated; pink, treated with plicamycin; red, treated with ML264; *n* = 15–20 mice per group). ns: *P* value > 0.05, ***P value ≤ 0.001, ###*P* value ≤ 0.001, †††*P* value ≤ 0.001. (C) Representative photographs of M-mode echocardiography of left ventricle in the section of the papillary muscle of the short axis. (D) Representative images of left ventricular muscle sections stained with wheat germ agglutinin (WGA) to measure cardiomyocyte size. Scale bar, 50 μm. (E) Bar plot showing the quantification of the myocyte cross-sectional area of the indicated groups based on WGA staining (*n* = 3 to 4 mice per group). ***P value ≤ 0.001. (F) Representative Masson’s trichrome staining images of heart sections in four groups to detect fibrosis. Scale bar, 500 μm. (G) Bar plot showing the quantitative analysis of myocardial fibrosis area based on Masson’s trichrome staining (*n* = 6 mice per group). **P value ≤ 0.01, ***P value ≤ 0.001. Data in (B, E, G) was expressed as mean ± SEM, and statistical significance was performed by two-tailed Student’s *t* test.

We generated a knock-in mouse model (*Myh6*^*R454C*/+^*Tnnt2*^*R127W/+*^) carrying two variants, p.R454C of *Myh6* (NM_010856.4) and p.R127W of *Tnnt2* (NM_001130179.2), which are orthologous to the pathogenic mutations identified in human HCM patients, p.R453C in *MYH7* and p.R102W in *TNNT2*, respectively (Fig. S6F, see Methods). We treated HCM mice with the SP1 inhibitor plicamycin or the EGR1 inhibitor ML264 to determine whether the HCM phenotypes could be alleviated (Fig. 5A, see Methods). Before injection (0 W), echocardiographic measurements revealed that the thickness of left ventricular posterior wall in diastole (LVPWd) was significantly greater in the three groups of HCM mice than in the wild-type (WT) mice (Fig. 5B). Strikingly, plicamycin or ML264 treatment for 4 or 6 weeks significantly alleviated the HCM phenotypes, as shown by a marked reduction in LVPWd thickness in the treated HCM mice compared to that in the untreated HCM mice (Fig. 5B and 5C). Consistently, compared with untreated HCM mice, HCM mice treated with plicamycin or ML264 exhibited a significant decrease in the cardiomyocyte cross-sectional area (Fig. 5D and 5E) and a reduction in myocardial fibrosis (Fig. 5F and 5G). These results demonstrated that the inhibition of TFs, which are activated via increased chromatin accessibility, can alleviate the development of HCM in mice.

### Plicamycin and ML264 reverse fetal gene reprogramming in HCM mice

**Figure 6.**
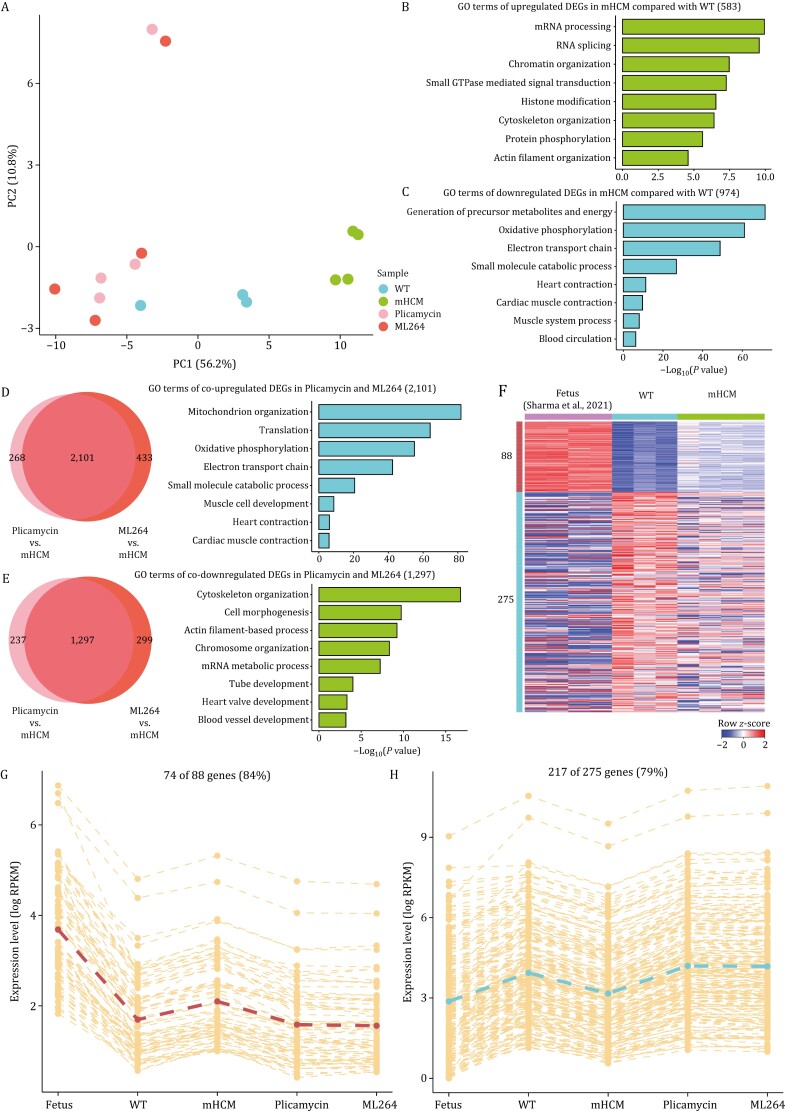
Transcriptomics analysis of WT mice, HCM mice, HCM mice treated with plicamycin or ML264, and fetal mice. (A) PCA plot showing the transcriptome pattern of protein-coding genes in WT mice, HCM mice, and HCM mice treated with plicamycin or ML264. PC1 and PC2 had variance values of 56.2% and 10.8%, respectively. (B) GO terms of 583 upregulated DEGs in HCM mice compared with WT. (C) GO terms of 974 downregulated DEGs in HCM mice compared with WT. (D) Venn diagrams showing the number of overlapping upregulated DEGs between the comparisons of HCM mice treated with plicamycin or ML264 vs. HCM mice. GO terms of 2,101 co-upregulated DEGs were shown on the right. (E) Venn diagrams showing the number of overlapping downregulated DEGs between the comparisons of HCM mice treated with plicamycin or ML264 vs. HCM mice. GO terms of 1,297 co-downregulated DEGs were shown on the right. (F) Heatmap showing row z-score scaled gene expression levels of co-upregulated and co-downregulated DEGs in both HCM mice and fetuses compared with WT mice. (G) Line plot showing the expression levels of 74 genes that co-upregulated in both HCM mice and fetuses compared with WT mice and co-downregulated in both HCM mice treated with plicamycin and ML264 compared with HCM mice. Gene expression levels were quantified with log_2_(RPKM + 1). (H) Line plot showing the expression levels of 217 genes that co-downregulated in both HCM mice and fetuses compared with WT mice and co-upregulated in both HCM mice treated with plicamycin and ML264 compared with HCM mice.

To explore the potential mechanism involved in the repression of HCM through the inhibition of SP1 and EGR1 with plicamycin and ML264, we performed RNA-seq using heart tissue samples collected from 4 groups of mice (WT mice, HCM mice, HCM mice treated with plicamycin or ML264). PCA clearly separated the untreated HCM mice from the others (Fig. 6A), which suggested that the transcriptomes of the HCM mice treated with plicamycin or ML264 were more similar to those of the WT mice than those of the untreated mutants. Compared with WT mice, 583 upregulated DEGs related to mRNA processing, chromatin organization, and actin filament organization were identified in the untreated HCM mice (Fig. 6B; Table S7). In addition, 974 downregulated DEGs were identified in the HCM mice, which were mainly enriched in the GO terms oxidative phosphorylation, electron transport chain, and heart contraction (Fig. 6C; Table S7). Compared with the untreated HCM mice, 2,369 and 2,534 upregulated DEGs were identified in the HCM mice treated with plicamycin and ML264, respectively (Table S8). Among these DEGs, 2,101 genes overlapped and were enriched in oxidative phosphorylation, electron transport chain and heart contraction (Fig. 6D). Notably, these GO terms were similar to those of the downregulated DEGs in the untreated HCM mice compared with the WT mice (Fig. 6C). Compared with the untreated HCM mice, 1,534 and 1,596 DEGs were downregulated in HCM mice treated with plicamycin or ML264, respectively (Table S8). Among these DEGs, 1,297 genes overlapped, which were enriched in GO terms such as actin filament-based process, chromosome organization, and mRNA metabolic process (Fig. 6E). Additionally, these GO terms were similar to those of the upregulated DEGs in the untreated HCM mice compared with the WT mice (Fig. 6B).

To investigate whether SP1 or EGR1 inhibition blocked fetal reprogramming of gene expression in HCM, we first identified the gene set that returned to the fetal pattern of expression in the HCM mouse model. We integrated the RNA-seq data of the left ventricle from mouse fetuses in a previous study (Sharma et al., 2021). In total, 88 DEGs were upregulated in both the fetal mice and the HCM mice compared with the WT mice (i.e., co-upregulated); in addition, 275 co-downregulated DEGs were identified (Fig. 6F). These 363 DEGs were considered to constitute the profile of fetal gene reprogramming in our HCM mice (Table S9). Among these DEGs, 74 of 88 (84%) co-upregulated DEGs and 217 of 275 (79%) co-downregulated DEGs were reversed in both of the two drug-treated groups (Fig. 6G and 6H; Table S9). These results indicated that plicamycin and ML264 can reverse fetal gene reprogramming in HCM at the transcriptome level.

## Discussion

One of the most important and crucial goals related to HCM is to understand the detailed mechanisms that mediate the process of pathological cardiac remodeling triggered by sarcomere mutations. This study constructed a multi-omics map of the HCM heart using NOMe-seq and RNA-seq, which included information on the transcriptome, DNA methylome, and chromatin accessibility at single-base resolution.

The reactivation of fetal genes in cardiac hypertrophy is an interesting phenomenon and is also referred to as the re-expression of a fetal gene program (Depre et al., 1998; Rajabi et al., 2007). Earlier studies have indicated reprogramming of fetal genes in the process of adaptation to heart stress, including structural protein isoform switching, the reactivation of protooncogenes, the induction of growth factors, and the expression of fetal isoforms of metabolic enzymes (Izumo et al., 1988; Nadal-Ginard and Mahdavi, 1989; Parker and Schneider, 1991; Razeghi et al., 2001). Our study showed a detailed comparison among HCM patients, healthy adults, and fetuses at multi-omics levels. In the transcriptome, we detected fetal gene reprogramming in the HCM myocardium, including decreased sarcomeric and metabolic gene expression and increased extracellular matrix gene expression (Figs. 4A–D, S5A and S5B). In the DNA methylome, the HCM hearts did not appear to be reprogrammed to a fetal state (Fig. S5C–F). The DNA methylome is more stable than the transcriptome and chromatin accessibility and is essential for maintaining genome stability (Robertson, 2005). With respect to chromatin accessibility, our results showed that the HCM hearts reverted to a fetal pattern at certain genomic regions (Figs. 4E, 4F, S5H and S5I), and the binding motifs of several TFs showed fetal-like enrichment patterns in HCM (Fig. 4G). In addition to gene expression, previous studies reported epigenetic control of fetal gene program, such as cardiac microRNAs and modifications of histone proteins, in the process of hypertrophic remodeling (Dirkx et al., 2013; Thum et al., 2007). These omics act on different levels of gene regulation and form a highly interlinked network. Multi-omics studies can more efficiently resolve this complicated network in HCM and offer candidate therapeutic strategies for this disease. Indeed, we found that the inhibition of SP1 and EGR1, which are newly found TFs with fetal chromatin accessibility patterns in patients, markedly repressed cardiac hypertrophy in HCM mice and thus provided potential drug targets for HCM treatment. The NAB1-EGR1 axis has been proved to be an important regulator of pathological cardiac growth (Buitrago et al., 2005). Interestingly, a recent study revealed that SP1 deficiency contributed to HCM in a mouse model (Zhang et al., 2024). However, many studies have demonstrated that SP1 is upregulated in pathological hypertrophy in different species (mouse, rat, and ewe) and models (Azakie et al., 2006a, 2006b; Lin et al., 2009; Long et al., 2020; Luo et al., 2019). Our study directly showed the upregulation of SP1 expression and an increase in chromatin accessibility in human HCM myocardium samples. Several studies have provided circumstantial evidence that blocking SP1 can inhibit extracellular matrix gene expression, suggesting that drugs targeting SP1 may be effective in treating fibro-proliferative diseases (Fajardo et al., 2011; Feng et al., 2023; Verrecchia et al., 2001).

Pharmacologic therapies recommended by guidelines for HCM remain palliative in fact, which focus on relieving symptoms due to HCM, such as first-line pharmacotherapy with β-blockers or nondihydropyridine calcium-channel blockers and second-line therapy with disopyramide (Tuohy et al., 2020; Wong and Martinez, 2019). These nonspecific pharmacotherapies are not designed for the treatment of HCM and have substantial side effects or limited evidence. Mavacamten is a first-in-class targeted inhibitor of cardiac myosin ATPase that shows great potential in obstructive hypertrophic cardiomyopathy (oHCM) by reducing cardiac contractility (Green et al., 2016; Ho et al., 2020). The development of therapies that directly target cardiac remodeling processes in HCM patients is urgently needed. Our data demonstrated the ability of plicamycin and ML264 to ameliorate the disease phenotype and delay disease progression in an HCM mouse model (Fig. 5B–G). Plicamycin, also called mithramycin A, is widely used as a selective inhibitor of SP1 as it can competitively interact with the GC-rich motif in promoters (Cao et al., 2024; Choi et al., 2014; Zou et al., 2024). Plicamycin is an anticancer drug that has been used for treating testicular carcinoma and myeloid leukemia prior to the current treatment regimen and shows potential in colorectal cancer cells (Deng et al., 2021; Dutcher et al., 1997; Kennedy and Torkelson, 1995; Quarni et al., 2019). During our *in vivo* experiment with plicamycin in an HCM mouse model, another team reported that the SP1 inhibitor plicamycin can protect cardiomyocytes from myocardial infarction (MI) *in vitro* (Geng et al., 2021), which, together with the results of the present study, suggest the great potential of applying plicamycin to treat heart diseases. ML264 is a small-molecule compound that inhibits the expression of EGR1 and its downstream transcription factor KLF5. ML264 might be a potential drug for treating colon cancer, osteosarcoma, and osteoarthritis (Huang et al., 2020; Ruiz de Sabando et al., 2016; Sun et al., 2019). Recently, a study showed that ML264 is useful for the treatment of ischemic heart failure in mice with MI (Hoffman et al., 2021). The two above examples indicate that reactivation of fetal genes is essential for HCM pathobiology and that reversing the fetal gene program may be a valuable therapeutic approach for HCM.

SP1 and EGR1 are widely expressed across various tissues and are crucial transcriptional factors in different biological processes such as cell proliferation and differentiation (Meriin et al., 2022; Vizcaíno et al., 2015; Wang et al., 2021; Xiao et al., 2023). Although apparent adverse effects were not noticed in the mice of our study, we speculate that ubiquitous inhibition of SP1 and EGR1 could result in impairment in organs beyond the heart. Furthermore, plicamycin and ML264, the widely used inhibitors of SP1 and EGR1, respectively, could have multifaceted actions beyond the inhibition of their targeted transcriptional factors. For instance, plicamycin not only prohibits the SP1 activity (Cao et al., 2024; Choi et al., 2014; Zou et al., 2024) but also affects the epigenetic modulators, including DNA methyltransferase and histone methyltransferase (Federico et al., 2020; Yuan et al., 2007). ML264 can affect the expression of KLF5 beside EGR1 (Huang et al., 2020; Ruiz de Sabando et al., 2016; Sun et al., 2019). Actually, plicamycin, when employed as an FDA-approved clinical chemotherapeutic agent, manifests several side effects, including hematological abnormalities, severe nausea, vomiting, diarrhea, and liver toxicity (Baum, 1968; Green and Donehower, 1984; Ream et al., 1968). In mice, it has been reported to induce liver toxicity characterized by high level of lipid accumulation and localized hepatocellular necrosis (Osgood et al., 2016). Interestingly, studies on ML264 have not explicitly mentioned side effects. Nevertheless, other research indicates that inhibiting EGR1 expression disrupts insulin gene expression, affecting insulin synthesis and resulting in abnormal glucose tolerance (Eto et al., 2006; Müller et al., 2012). Furthermore, EGR1 inhibition can impair reproductive function and hinder embryo implantation in mice (Guo et al., 2014). Thus, the clinical translation of our findings to HCM treatment might require precious targeted cardiac-deliver of more specific inhibitors of these transcription factors.

**Figure 7.**
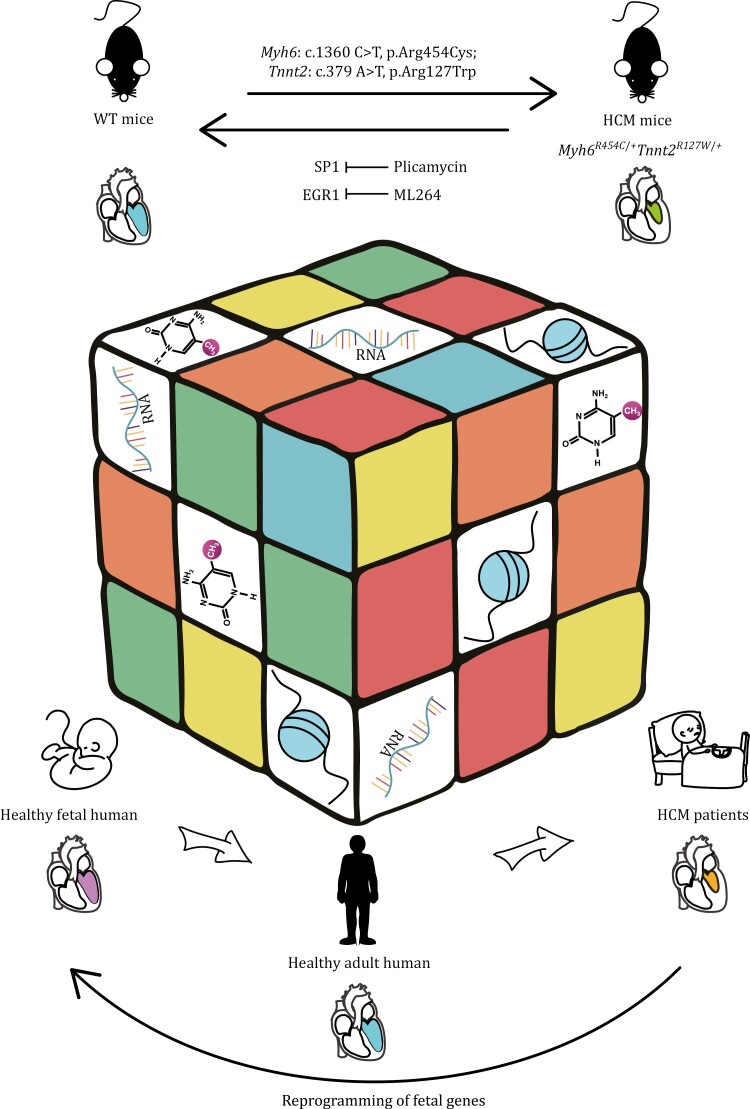
Summary diagram of multi-omics analysis in HCM. The magic cube shows that multi-omics (transcriptome, DNA methylome, and chromatin accessibility) form a highly interlinked network and play important but different roles together in cardiac development and pathology. The HCM heart demonstrates fetal gene reprogramming and inhibition of SP1 or EGR1 can alleviate the development of HCM in mice.

In conclusion, our study integrated transcriptome, DNA methylation, and chromatin accessibility data and provided insights into cardiac remodeling in HCM (Fig. 7). Through the establishment of high-precision multi-omics maps of HCM hearts, we identified potential key TFs for the reactivation of fetal genes in HCM, which may be potential drug targets for HCM treatment. In particular, we demonstrated that the SP1 inhibitor plicamycin and the EGR1 inhibitor ML264 had therapeutic effects on attenuating cardiac hypertrophy in an HCM mouse model, possibly by reversing fetal reprogramming. Together with our previous work (Gao et al., 2021), we constructed valuable resources that contain multi-omics and single-base resolution data for heart tissues from human adults and fetuses, the HCM patients, as well as WT and HCM mice.

## Methods

### Patient sample acquisition

HCM samples were obtained from 12 patients with obstructive HCM who underwent a Morrow septal myectomy at Fuwai Hospital. Key clinic parameters of HCM patients are listed in Table S1. The HCM diagnosis was based on a maximum left ventricular wall thickness of ≥15 mm detected by echocardiography and/or cardiac magnetic resonance without secondary hypertrophy caused by other cardiac or systemic diseases (e.g., cardiac valve disease and uncontrolled hypertension) and HCM phenocopies (Gersh et al., 2011). All tissue specimens were immediately collected and stored in liquid nitrogen until use.

### RNA extraction and RNA-seq library preparation from HCM patient samples

A small portion (20–30 mg) of each frozen myocardium sample from HCM patients was used for RNA isolation and RNA-seq library construction as described previously (Gao et al., 2021). In summary, myocardial sample homogenization was accomplished by grinding the tissue in liquid nitrogen and using the QIAshredder (QIAGEN, 79656). Total RNA from the hearts of HCM patients was extracted with the RNeasy Fibrous Tissue Mini Kit (QIAGEN, 74704) following the manufacturer’s instruction. We depleted rRNA from total RNA through the use of the NEBNext rRNA Depletion Kit (NEB, E7755X). Soon after, we synthesized first-strand cDNA through the NEBNext RNA First Strand Synthesis Module (NEB, E7525L) and second-strand through the NEBNext Ultra II Non-Directional RNA Second Strand Synthesis Module (NEB, E6111L). In the end, the RNA-seq library was constructed with KAPA Hyper Prep Kits (KAPA Biosystems, KK8504).

### NOMe-seq library construction of HCM patients

About 30 mg of heart tissue from each HCM patient was used for NOMe-seq library construction based on an optimal protocol we reported previously (Gao et al., 2021). In short, frozen myocardial samples from HCM patients were ground in liquid nitrogen. We resuspended the tissue pellets in 500 μL of ice-cold lysis buffer and incubated the samples for 1 h to release nuclei. After washing with cold DPBS twice and adding 3 ng of unmodified lambda DNA (Thermo Fisher Scientific, SD0021), the lysates were incubated with 60 U of GpC Methyltransferase, M.CviPI (NEB, M0227L) for 1 h and 20 U of supplementary M.CviPI for another hour to profile chromatin accessibility. We stopped the reactions by adding EDTA and digested histones overnight with proteinase K. Phenol:chloroform:isoamyl alcohol extraction and ethanol precipitation were applied to the purified genomic DNA. The genomic DNA was bisulfite converted by EZ-96 DNA Methylation-Direct MagPrep (Zymo Research, D5044) to obtain DNA methylation information. Whereafter, the first and second strands were synthesized using the random primers Oligo1 (5ʹ-biotin-CTACACGACGCTCTTCCGATCTNNNNNNNNN-3ʹ) and Oligo2 (5ʹ-AGACGTGTGCTCTTCCGATCTNNNNNNNNN-3ʹ), respectively. The final NOMe-seq libraries were amplified via approximately 12 cycles of PCR in KAPA HiFi Hot Start Ready Mix (KAPA Biosystems, KK2602). Two technical replicates of one HCM patient were used to increase the reliability of NOMe-seq data.

### HCM mouse model construction and experiment

To mimic disease in animals, we generated a knock-in mouse with two causal mutations of HCM: c.1360 C > T (p.Arg454Cys, NM_010856.4) of *Myh6* in mouse which is orthologous to c.1357 C > T (p.Arg453Cys, NM_000257.4) of *MYH7* in human, and c.379 A > T (p.Arg127Trp, NM_001130179.2) of *Tnnt2* in mouse which is orthologous to c.304 C > T (p.Arg102Trp, NM_000364.4) of *TNNT2* in human. We first constructed two mouse lines carrying each of the two mutations via CRISPR/Cas9-mediated targeted integration on a C57BL/6J genetic background at the Institute of Medical Laboratory Animals, Chinese Academy of Medical Sciences. The designed sgRNAs for the introduction of the two mutations targeted the sequence CCG CGCCAGTACTTCATAGGTGT (underscored, PAM) on exon 13 of mouse *Myh6* and ACATCCACAGGAAGCGCG TGG (underscored, PAM) on exon 9 of mouse *Tnnt2.* After *in vitro* transcription, the Cas9 mRNA and sgRNA were microinjected into the pronuclei of fertilized mouse eggs (C57BL/6J) and then transferred into pseudopregnant female foster mice to produce offspring. The transgenic progeny were ultimately validated via Sanger sequencing after PCR amplification of genomic DNA isolated from mouse tail tips. The heterozygous mice with each of the two mutations were then crossed with each other to produce mice with both *Myh6* and *Tnnt2* heterozygous mutations (*Myh6*^*R454C*/*+*^*Tnnt2*^*R127W*/+^), which were used as HCM model mice. WT littermates were used as controls in further experiments. Four-week-old HCM mice were randomly divided into three groups and intraperitoneally injected with plicamycin (0.2 mg/kg/day) (MCE, HY-A0122), ML264 (20 mg/kg/2 day) (MCE, HY-19994) or vehicle (10% DMSO + 40% PEG300 + 5% Tween 80 + 45% saline). WT mice were also intraperitoneally injected with vehicle. Animals were raised in a constant temperature and humidity barrier system with a 12 h light-dark cycle and received water and food ad libitum using a standard chow diet.

### RNA extraction and RNA-seq library preparation of the HCM mouse model

Total RNA was extracted from the four groups using TRIzol reagent (Invitrogen, 15596018) according to the manufacture’s instruction: wild-type mouse (*n* = 3 male mice), mouse HCM model (*n* = 4 male mice), mouse HCM model treated with plicamycin (*n* = 4 male mice), or ML264 (*n* = 4 male mice). After RNA extraction, the mouse RNA-seq library construction was the same as that used for human HCM. RNA-seq data of the left ventricle at E14 of fetal mice were downloaded for integrated analysis (Sharma et al., 2021). The NOMe-seq and RNA-seq libraries of HCM patients and mouse models were sequenced on the Illumina HiSeq 4000 platform in 150 bp paired-end mode (Novogene).

### Echocardiography

Cardiac function was determined by echocardiography (Visual Sonics, Vevo 2100, 40 MHz 550 probe). Echocardiography was performed at 0, 4, and 6 weeks after plicamycin or ML264 injection. Mice (*n* = 15–20 per group) were anesthetized in an induction chamber with 3% isoflurane and maintained in an anesthetized state with 0.5%–1% isoflurane on a temperature-controlled plate (37°C). M-mode recordings were obtained from a short-axis view at the level of the papillary muscles to assess left ventricular function. The left ventricular chamber size and wall thickness were measured using Vivo Lab 3.1 software (Visual Sonic). The left ventricular posterior wall in diastole (LVPWd) was selected to assess left ventricular hypertrophy. All parameters were measured for at least three beats independent of respiration from each projection and averaged.

### Histologic analysis

Mouse hearts were harvested and then fixed in 10% neutral formalin for 24 h at room temperature. All tissues were dehydrated with a series of ethanol and dimethylbenzene solutions and then embedded in paraffin. The tissue was sliced into 5 μm thick sections. We stained heart sections with standard Masson trichrome stain (Sigma, HT15-1KT) to assess myocardial fibrosis. The degree of fibrosis was quantified with ImageJ software, and the fibrosis ratio was calculated by evaluating the fibrotic area and total area of each cross-section. To determine the cross-sectional area (CSA) of cardiomyocytes, we stained heart sections with Oregon Green 488-conjugated wheat germ agglutinin (WGA; Thermo Fisher Scientific, W6748). For each left ventricle tissue sample, 7 images were selected for the statistical analysis of cardiac myocyte CSA. Twenty to thirty round-to-ovoid cells were selected from each image and analyzed with ImageJ software (NIH).

### Real-time quantitative PCR (RT-qPCR)

Reverse transcription was performed on 1 μg of total RNA using PrimeScript RT Master Mix (Takara, RR047A). Using cDNA as the template, RT-qPCR was performed in triplicate using SYBR Green qPCR Master Mix (Takara, RR820A) with a Vii7 Real-Time PCR System (Applied Biosystems). The 2^−ΔΔCt^ method was used to determine the relative gene expression. The specific primers used were as follows: human *SP1*, forward primer 5ʹ-CCCTTGAGCTTGTCCCTCAG-3ʹ and reverse primer 5ʹ-TGAAAAGGCACCACCACCAT-3ʹ; human *EGR1*, forward primer 5ʹ-CTTCAACCCTCAGGCGGACA-3ʹ and reverse primer 5ʹ-GTTTGGCTGGGGTAACTGGT-3ʹ; and human *GAPDH*, forward primer 5ʹ-ACAACTTTGGTATCGTGGAAGG-3ʹ and reverse primer 5ʹ-GCCATCACGCCACAGTTTC-3ʹ.

### Western blot

Proteins from heart tissues were extracted using RIPA lysis buffer (Beyotime, P0013B) supplemented with protease and phosphatase inhibitors (Roche, 5892791001 and 49068450001). The protein concentration was determined using a BCA protein quantification kit (Thermo Fisher Scientific, 23227). A total of 10 μg of protein was resolved on a 4%–12% precast SDS-polyacrylamide electrophoresis gel (Invitrogen, NP0322BOX). Afterward, the SDS gels were transferred to PVDF membranes (Merck Millipore, IPVH00010), and the membranes were blocked with 5% nonfat milk (CST, 9999S) for 2 h at room temperature. Next, the membranes were incubated with primary antibodies at 4°C overnight with mild shaking. After the membranes had been washed four times with Tris-buffered saline with Tween 20 (TBST) for 5 min each, they were then incubated for 1 h with secondary antibodies. The following antibodies were used for Western blot: anti-SP1 (Proteintech, 21962-1-AP, 1:2,000); anti-EGR1 (Santa Cruz Biotechnology, sc-101033, 1:1000); anti-GAPDH (Proteintech, 60004-1-Ig, 1:20,000); Goat anti-Mouse IgG (H + L) Cross-Adsorbed Secondary Antibody, HRP (Invitrogen, G21040, 1:20,000); Goat anti-Rabbit IgG (H + L) Secondary Antibody, HRP (Invitrogen, 31460, 1:20,000). Chemical luminescence from the membranes was measured with a chemiluminescence image analysis system (Tianneng) and visualized with a Super Signal West Femto chemiluminescence kit (Thermo Fisher Scientific, 34096).

### RNA-seq data processing

The raw data were first processed to remove reads with low-quality bases and trim adaptors. Then, the clean reads were mapped to the GENCODE human genome (hg19) or mouse genome (mm10) using TopHat (version: 2.0.12) with the default parameters (Trapnell et al., 2009). HTSeq (version: 0.11.1) was used to count reads, and the gene expression levels were estimated using the RPKM method (reads per kilobase transcriptome per million reads).

### Principal component analysis (PCA) and hierarchical clustering

PCA was performed using the prcomp function in the R package stats in Bioconductor (Gentleman et al., 2004; Huber et al., 2015) and the PCA function in the R package pcaMethods (Stacklies et al., 2007). Pearson coefficients were computed, and hierarchical clustering was performed using the “hclust” function in the R package stats with “ward.D2” method.

### Identification of differentially expressed genes

DEGs were determined using the R package DESeq2 (version: 1.32.0) (Love et al., 2014). Protein-coding RNAs meeting the following criteria were considered differentially expressed coding genes: (1) absolute log_2_-transformed fold change > 0.5 and FDR ≤ 0.05 (Benjamini and Hochberg); and (2) average RPKM greater than 1. LncRNAs matching the following 2 criteria were considered DEGs: (1) absolute log_2_-transformed fold change > 0.5 and FDR ≤ 0.05; and (2) average RPKM greater than 0.1. GO analysis was performed using ToppGene (Chen et al., 2009), and significantly enriched GO terms from biological process were selected.

### Annotation of *cis*-regulatory relationships between lncRNAs and protein-coding genes

The Pearson correlation between lncRNAs and protein-coding genes was calculated for DEGs between the HCM and normal groups to detect co-expressed gene pairs. There were 691 up-regulated differentially expressed protein-coding genes, 207 up-regulated differentially expressed lncRNAs, 835 down-regulated differentially expressed protein-coding RNAs and 264 down-regulated differentially expressed lncRNAs in the HCM group. Co-expressed gene pairs were considered to have a *cis*-regulatory relationship only if they met the following criteria: (1) a Pearson correlation coefficient ≥ 0.6 and a Pearson correlation test *P* value ≤ 0.05; and (2) a linear distance between the lncRNA and protein-coding RNA in the co-expressed gene pair within 100 kb.

### NOMe-seq data processing

The NOMe-seq reads were trimmed with Trim Galore (version: 0.3.3) to remove random primer sequences, adaptors and low-quality bases with the parameters “—quality 20—stringency 3—length 50—clip_R1 9—clip_R2 9—paired—trim1—phred33—gzip”. The clean reads were aligned to the UCSC human genome (hg19) using Bismark (version: 0.7.6) (Krueger and Andrews, 2011) in paired-end and non-directional mode, and then, the unmapped reads were realigned to the same reference genome in single-end and non-directional mode. PCR duplications were removed by SAMtools (version: 0.1.18) (Li et al., 2009).

### Determination of DNA methylation and chromatin accessibility levels

The methylation level of each covered cytosine site was calculated by the ratio of the number of methylated reads “C” divided by the number of methylated and unmethylated reads (“C + T”). GCG and CCG trinucleotides were excluded in the downstream analysis. GCG trinucleotides were removed to avoid confusion between DNA methylation and chromatin accessibility, and CCG trinucleotides were also removed because M.CviPI methyltransferase has slight activity for CC sites (Kelly et al., 2012). As a result, we used WCG (W represents A or T nucleotides) for DNA methylation analysis and GCH (H denotes A, T or C nucleotides) for chromatin accessibility analysis.

The 3× coverage was adopted as the read depth cutoff in the subsequent analysis. DNA methylation levels and chromatin accessibility levels were estimated for each genomic region, and only regions with at least three WCG/GCH sites were retained.

The whole human genome was divided into bins with a size of 1 kb. The annotations of exon, intron, CpG island (CGI), transcription start site (TSS), transcription end site (TES), and repeat region were downloaded from the UCSC genome browser (hg19). The gene bodies and intragenic regions were defined as the regions from the TSS to the TES, while intergenic regions were considered the complementary regions of the intragenic regions in human genome. The promoter was defined as the region 1 kb upstream of the TSS and 0.5 kb downstream of the TSS. Human enhancer information was collected from the “ENCODE cCREs” track from the UCSC Genome Browser (hg38), and then, we converted the genomic coordinates of enhancers from hg38 to hg19 using the chain file and the liftOver method from the UCSC Genome Browser.

WCG levels were observed around gene bodies, each gene body was divided into 100 bins, and extensions of the gene body (2 kb upstream of the TSS and 2 kb downstream of the TES) were separated into 200 bins with a size of 20 bp. GCH levels were observed around 2 kb upstream and 2 kb downstream from the TSS, and each region was separated into 200 bins with a size of 20 bp.

### Identification of differentially methylated regions

DMRs were identified based on the 3× coverage WCG sites. The whole human genome was divided into 300-bp windows to calculate the average WCG level in each sample, and windows with at least 3 WCG sites were obtained. Differentially methylated windows (DMWs) between healthy controls and HCM patients fulfilled the following strict criteria: the average difference between two groups was more than 20%, with a two-tailed Student’s *t* test *P* value ≤ 0.05 and an FDR ≤ 0.05 (Benjamini and Hochberg). DMRs were merged from adjacent DMWs within 300 bp.

### Definition of nucleosome-depleted regions

NDRs were identified with 3× coverage GCH sites. The GCH methylation level of 100-bp windows with 20-bp sliding steps was calculated to call NDRs (Chen et al., 2020; Li et al., 2018), which are the regions with significantly higher GCH levels than the whole-genomic background. The regions that met the following criteria were considered NDRs: (1) the average GCH level of the region was significantly higher than the whole-genome background with a chi-squared test *P* value ≤ 10^−10^; (2) GCH sites ≥ 5; and (3) the length of the region was no less than 140 bp.

The common intervals among NDRs from corresponding groups were identified by the “bedtools multiinter” command of bedtools (v2.28.0), and then, neighboring NDRs within 10 bp were connected using the “bedtools merge” command of bedtools (Quinlan and Hall, 2010). NDRs were divided into two groups based on their distance from the TSS: NDRs located within promoters were defined as proximal NDRs, and the others were defined as distal NDRs. When performing PCA based on the chromatin accessibility of the proximal or distal NDRs, NDRs from all samples were merged.

### Relating transcription factor binding motifs to chromatin accessibility

The NDRs were merged respectively in three groups: healthy adult controls, HCM patients, and fetuses. Transcription factor binding motif enrichment in NDRs was conducted using “findMotifsGenome.pl” in HOMER (version: 4.10.4) (Heinz et al., 2010) with the command “-size 2000 -len 8 -S 100”. Only motifs that met 2 criteria were retained for further analysis: (1) *P* value ≤ 10^−10^; and (2) RPKM ≥ 5 in at least 1 sample.

## Supplementary data

The online version contains supplementary material available at https://doi.org/10.1093/procel/pwae032.

pwae032_suppl_Supplementary_Materials

## Data Availability

The authors confirm that all data underlying the findings are fully available without restriction. The raw RNA-seq and NOMe-seq data of HCM patients have been deposited in the Genome Sequence Archive (GSA) for Human under the accession code HRA002044. And the raw RNA-seq of the HCM mouse model has been deposited in GSA under CRA011896. The processed RNA-seq data (gene expression matrices) of humans and mice have been deposited in OMIX under the IDs OMIX004687 and OMIX004688, respectively. The processed NOMe-seq data (bed files) of humans were deposited in OMIX under the IDs OMIX004694 (WCG for DNA methylation analysis) and OMIX004695 (GCH for chromatin accessibility analysis). All the above-mentioned data can be accessed in GSA under BioProject ID PRJCA007579. Requests for materials should be addressed to Jizheng Wang, Lu Wen, Lei Song, or Shuiyun Wang.

## Code availability

Codes are available from the corresponding authors upon reasonable request.
