# Supplementary material for: Integrative analysis of transcriptome, DNA methylome, and chromatin accessibility reveals candidate therapeutic targets in hypertrophic cardiomyopathy
Source: Protein Cell. 2024 May 23;15(11):796–817. doi: 10.1093/procel/pwae032 (PMC11528543; doi:10.1093/procel/pwae032)
Supplement: pwae032_suppl_Supplementary_Materials [file pwae032_suppl_supplementary_materials.zip › PAC-24076-WL-Supplmentary Materials/PAC-24076-WL-Supplmentary Materials.pdf]

Supplemental figure legends

Fig.S1. Differentially expressed protein-coding genes between HCM patients and healthy donors.

Dark goldenrod dots were up-regulated DEGs with FDR < 0.05 and $\log_2$(Fold Change) > 0.5; cyan dots were down-regulated DEGs with FDR < 0.05 and $\log_2$(Fold Change) < -0.5. Squares meant that these genes met the criteria: FDR < 10^{-30} or FDR < 10^{-10} and absolute value of $\log_2$(Fold Change) was greater than 4.

Fig.S2. The comparison of HCM patients and healthy controls at endogenous DNA methylation levels.

(A) Line plot showing the average endogenous DNA methylation levels in the gene body and 2 kb upstream and downstream regions of gene body in HCM patient ind110. Different colors indicated different technology replications.

(B) PCA plot showing the endogenous DNA methylation pattern of the whole genome in HCM patients and healthy controls. PC1 and PC2 had variance values of 4.3% and 3.8%, respectively.

(C) Bar plots showing the numbers of hypermethylated and hypomethylated DMRs located in different genome elements.

(D) Bar plot showing the average endogenous DNA methylation levels in different genome elements. ns: *P* value > 0.05.

(E) PCA plot showing the endogenous DNA methylation pattern of gene-bodies in HCM patients and controls. PC1 and PC2 had variance values of 10.1% and 4.6%, respectively.

(F) PCA plot showing the endogenous DNA methylation pattern of CpG Islands in HCM patients and controls. PC1 and PC2 had variance values of 9.4% and 6.7%, respectively.

(G) Heatmap showing endogenous DNA methylation levels in the gene body ± 10 kb regions of *ACTA1* in all HCM patients and healthy controls. The gene expression levels of *ACTA1* were showed in the boxplot on the right, and the gene expression levels were quantified by $\log_2$(RPKM +1). The color bars in the heatmap represented the regions of gene body and promoter (from 1 kb upstream of the TSS to 0.5 kb downstream of the TSS), and the direction of the arrow indicated the direction of transcription. ns: *P* value > 0.05.

(H) IGV image showing the endogenous DNA methylation levels of *ACTA1* and adjacent regions in 4 HCM patients and 4 controls. Each vertical line represented a site, and the height of the vertical line represented the endogenous DNA methylation level. DMR_NC and DMR_NHCM represent the hypomethylated DMRs and hypermethylated DMRs identified in HCM patients, respectively. ENCODE_cCRE_Hg38ToHg19 showed the position of the enhancers. Statistical significance was performed by two-tailed Student *t* test.

Fig.S3. The changes of chromatin accessibility between HCM patients and controls.

(A) Line plot showing the average chromatin accessibility levels in the 2 kb upstream and downstream regions of TSS in HCM patient ind110. Different colors indicated different technology replications.

(B) Boxplot showing the average chromatin accessibility levels in the proximal and distal NDRs in HCM patients and healthy adult controls. ns: *P* value > 0.05, ***: *P* value < 0.001.

(C) PCA plot showing the chromatin accessibility pattern of proximal NDRs in HCM patients and

controls. PC1, PC2 and PC3 had variance values of 9.7%, 4.3% and 3.9%, respectively.

(D) PCA plot showing the chromatin accessibility pattern of distal NDRs in HCM patients and controls. PC1, PC2 and PC3 had variance values of 5.9%, 3.7% and 3.5%, respectively.

(E) PCA plot showing the chromatin accessibility pattern of promoters in HCM patients and healthy donors. PC1 and PC2 had variance values of 18.5% and 6.2%, respectively.

(F) PCA plot showing the chromatin accessibility pattern of gene-bodies in HCM patients and healthy donors. PC1 and PC2 had variance values of 24.9% and 5.1%, respectively.

(G) PCA plot showing the chromatin accessibility pattern of enhancers in HCM patients and healthy donors. PC1 and PC2 had variance values of 6.6% and 3.5%, respectively. Statistical significance was performed by two-tailed Student *t* test.

Fig.S4. Correlations among transcriptome, DNA methylome, and chromatin accessibility in HCM patients and healthy controls.

(A) Line plots showing the average endogenous DNA methylation level in the gene body ± 2 kb regions of protein-coding genes in controls and HCM patients. Different colors represented 4 gene groups classified by gene expression levels. Silenced genes: RPKM $\leq$ 0.1; low-expression genes: 0.1 $<$ RPKM $\leq$ 1; intermediate-expression genes: 1 $<$ RPKM $\leq$ 10; high-expression genes: RPKM ＞ 10.

(B) Line plots showing the average chromatin accessibility level in the TSS ± 2 kb regions of protein-coding genes in controls and HCM patients. Different colors represented 4 gene groups classified by gene expression levels.

(C) Line plots showing the average endogenous DNA methylation levels (green) and the average chromatin accessibility levels (blue) in the promoters of corresponding genes. Genes were ranked by expression levels (red), and the x-axis from left to right indicated genes with increased expression levels. R1 indicated spearman correlation coefficients between endogenous DNA methylation levels and gene expression levels and R2 indicated spearman correlation coefficients between chromatin accessibility levels and gene expression levels.

(D) Line plots showing the average endogenous DNA methylation levels of the gene bodies in each bin. Genes (RPKM > 0) were ranked by expression levels and grouped into 100 bins, and the x-axis from left to right indicated genes with increased expression levels. Each point indicated a bin. The shadow represented the 0.95 confidence interval around smooth.

(E) Line plots showing the average chromatin accessibility level of the gene bodies in each bin.

Fig.S5. Multi-omics analysis of the adult controls, HCM patients and fetuses.

(A) Heatmap showing row z-score scaled gene expression levels of co-upregulated and co-downregulated DEGs (protein-coding genes) in both HCM patients and fetuses compared with adult controls.

(B) GO terms of corresponding co-regulated DEGs.

(C) Violin plot showing the genome-wide endogenous DNA methylation levels (left) and line plot showing the average endogenous DNA methylation levels in the gene body and 2 kb upstream and downstream regions of gene body in the adult controls, HCM patients and fetuses (right). ns: P > 0.05.

(D) PCA plot showing the DNA methylation pattern of promoters in healthy donors, HCM patients and fetuses. PC1 and PC2 had variance values of 8.4% and 5.7%, respectively.

(E) PCA plot showing the DNA methylation pattern of gene-bodies in three groups. PC1 and PC2 had variance values of 11.3% and 7.8%, respectively.

(F) PCA plot showing the DNA methylation pattern of enhancers in three groups. PC1 and PC2 had variance values of 12.7% and 3.7%, respectively.

(G) Violin plot showing the total accessibility levels of whole genome (left) and line plot showing the average chromatin accessibility levels in the 2 kb upstream and downstream regions of TSS in the adult controls, HCM patients and fetuses (right). ns: P > 0.05.

(H) PCA plot showing the chromatin accessibility pattern of distal NDRs in three groups. PC1 and PC2 had variance values of 5.6% and 3.2%, respectively.

(I) Unsupervised hierarchical clustering analysis of chromatin accessibility of distal NDRs in the adult controls, the HCM patients and the fetuses. Statistical significance was performed by two-tailed Student *t* test.

Fig.S6. The expression of SP1 and EGR1 in the adult controls, patients with HCM and fetus.

(A) Boxplot showing the expression levels of *SP1* in healthy controls, HCM patients and fetuses. Gene expression levels were quantified with $\log_2$(RPKM + 1).

(B) Boxplot showing the expression levels of *EGR1* in healthy controls, HCM patients and fetuses.

(C) Bar plot showing the relative expression of *SP1* in controls and HCM patients by RT-qPCR. Values were normalized to *GAPDH* expression (n = 6 people in controls and n = 15 people in HCM patients).

(D) Bar plot showing the relative expression of *EGR1* in controls and HCM patients by RT-qPCR. Values were normalized to *GAPDH* expression (n = 6 people in controls and n = 15 people in HCM patients).

(E) Western blot (left) and bar plots (right) showing SP1 and EGR1 protein levels in controls and HCM patients (n = 4 people per group).

(F) The schematic diagram of breeding strategy for HCM mice carrying *Myh6* (c.1360 C>T) and *Tnnt2* (c.379 A>T) mutations. Mice with $Myh6^{R454C/+}$ or $Tnnt2^{R127W/+}$ mutation were constructed, and the double-mutant HCM mice model ($Myh6^{R454C/+}Tnnt2^{R127W/+}$) was generated by cross-breeding between the two genotypes. Data in (C, D, E) were expressed as mean ± SEM, and statistical significance was performed by two-tailed Student *t* test. ns: *P* value > 0.05, *: *P* value ≤ 0.05, **: *P* value ≤ 0.01, ***: *P* value ≤ 0.001.

Fig S1

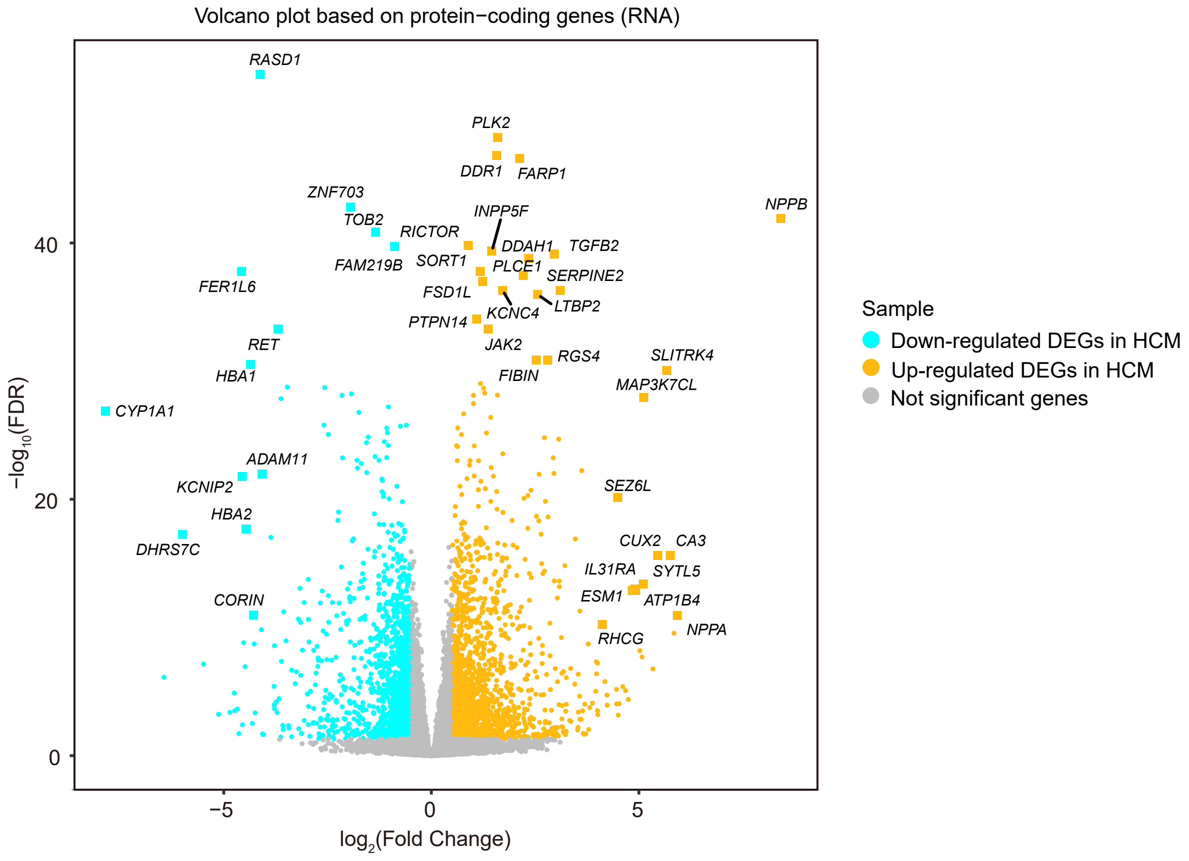

Volcano plot based on protein−coding genes (RNA)
RASD1
PLK2
DDR1
FARP1
ZNF703
TOB2
INPP5F
NPPB
RICTOR
DDAH1
TGFB2
FAM219B
SORT1
PLCE1
SERPINE2
FER1L6
FSD1L
KCNC4
LTBP2
PTPN14
RET
JAK2
RGS4
SLITRK4
HBA1
FIBIN
MAP3K7CL
CYP1A1
ADAM11
KCNIP2
SEZ6L
HBA2
CUX2
CA3
DHRS7C
IL31RA
SYTL5
ESM1
ATP1B4
CORIN
RHCG
NPPA
−log10(FDR)
log2(Fold Change)
Sample
Down-regulated DEGs in HCM
Up-regulated DEGs in HCM
Not significant genes

Fig S2

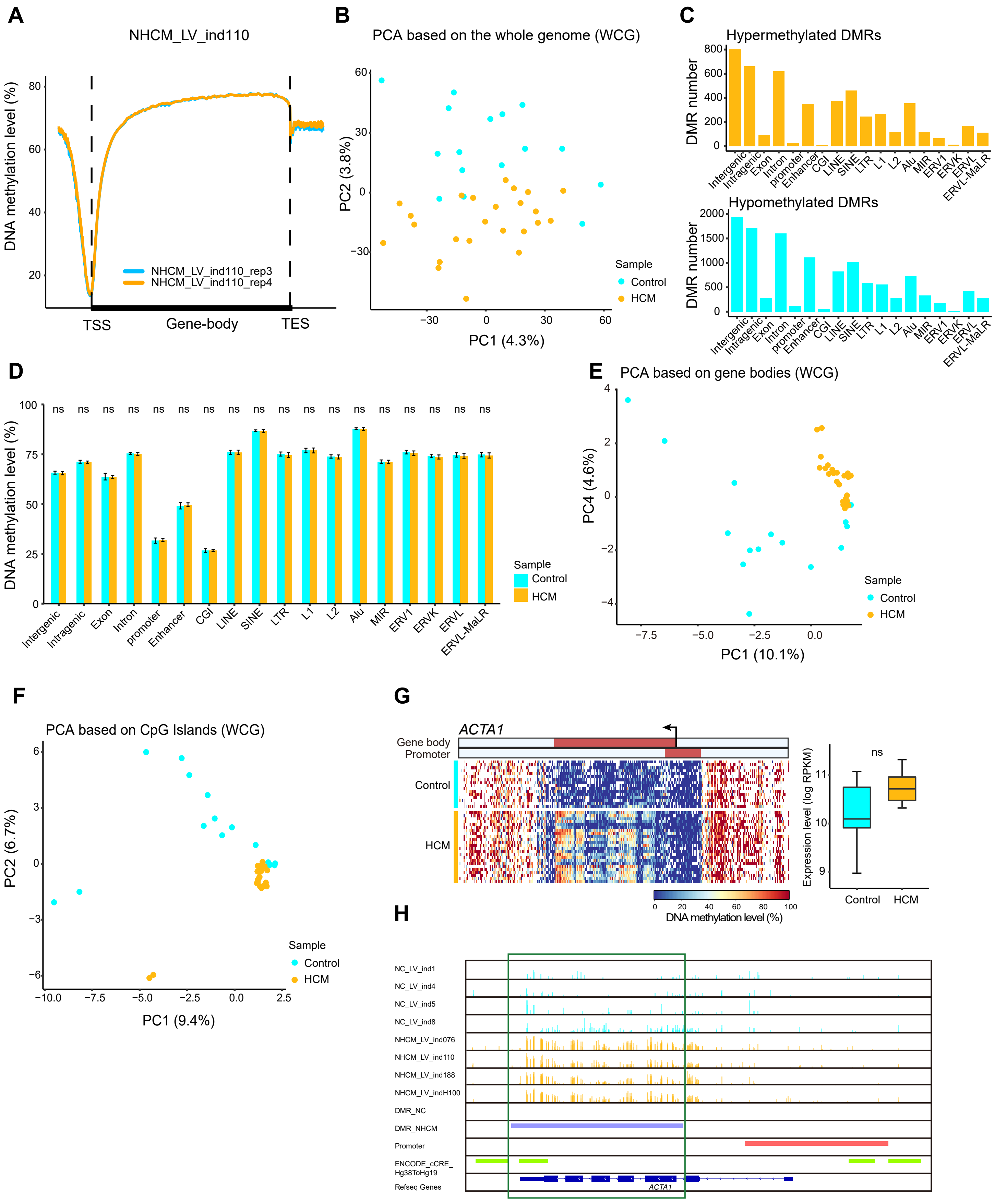
A
NHCM_LV_ind110
DNA methylation level (%)
NHCM_LV_ind110_rep3
NHCM_LV_ind110_rep4
TSS
Gene-body
TES
B
PCA based on the whole genome (WCG)
PC1 (4.3%)
PC2 (3.8%)
Sample
Control
HCM
C
Hypermethylated DMRs
Hypomethylated DMRs
DMR number
Intergenic
Intragenic
Exon
Intron
promoter
Enhancer
CGI
LINE
SINE
LTR
L1
L2
Alu
MIR
ERV1
ERVK
ERVL
ERVL-MaLR
D
DNA methylation level (%)
ns
Sample
Control
HCM
E
PCA based on gene bodies (WCG)
PC1 (10.1%)
PC4 (4.6%)
F
PCA based on CpG Islands (WCG)
PC1 (9.4%)
PC2 (6.7%)
G
ACTA1
Gene body
Promoter
Control
HCM
DNA methylation level (%)
Expression level (log RPKM)
ns
H
NC_LV_ind1
NC_LV_ind4
NC_LV_ind5
NC_LV_ind8
NHCM_LV_ind076
NHCM_LV_ind110
NHCM_LV_ind188
NHCM_LV_indH100
DMR_NC
DMR_NHCM
Promoter
ENCODE_cCRE_
Hg38ToHg19
Refseq Genes
ACTA1

Fig S3

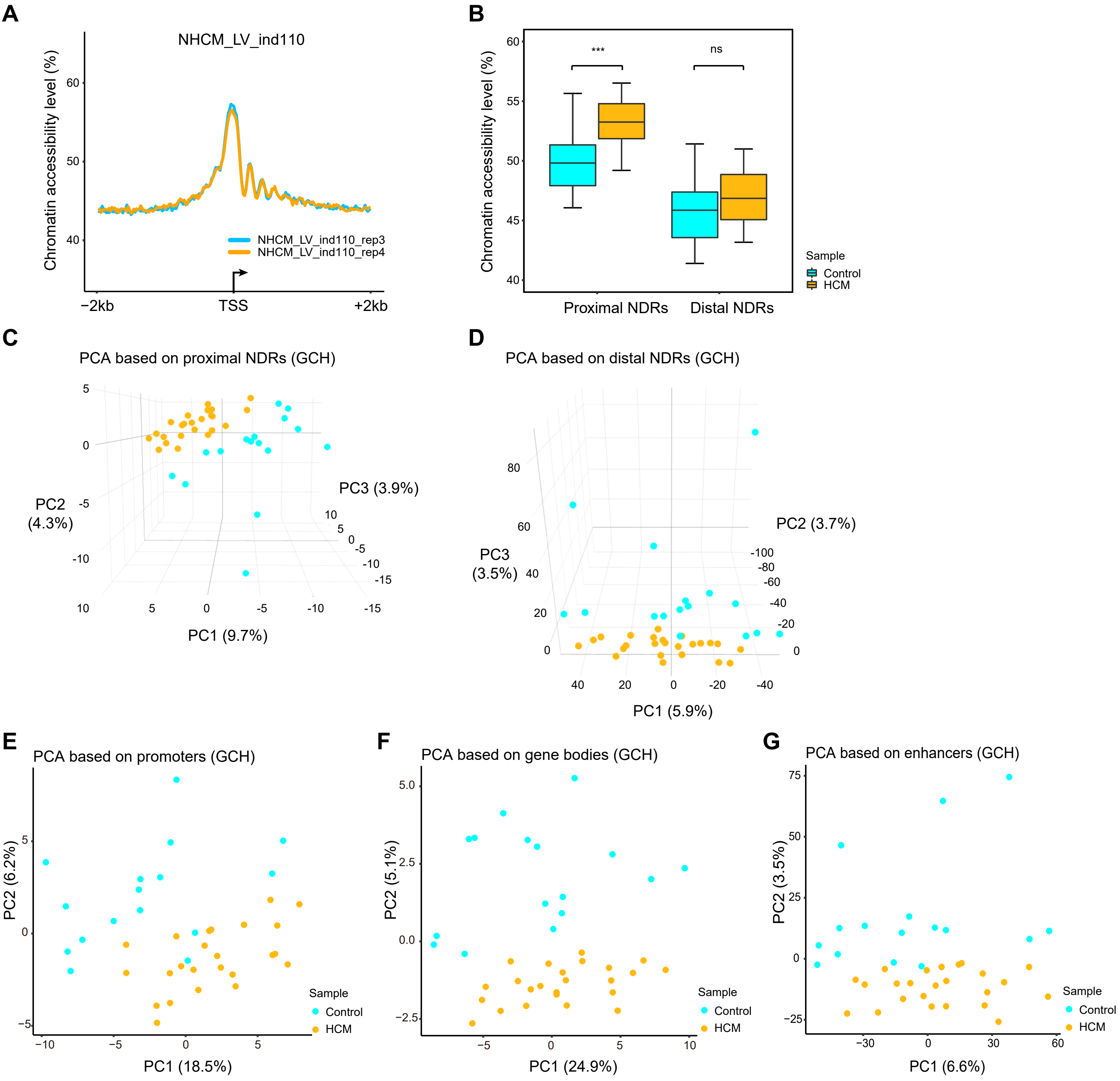
A
NHCM_LV_ind110
Chromatin accessibility level (%)
NHCM_LV_ind110_rep3
NHCM_LV_ind110_rep4
−2kb
TSS
+2kb
B
Chromatin accessibility level (%)
***
ns
Proximal NDRs
Distal NDRs
Sample
Control
HCM
C
PCA based on proximal NDRs (GCH)
PC2 (4.3%)
PC3 (3.9%)
PC1 (9.7%)
D
PCA based on distal NDRs (GCH)
PC3 (3.5%)
PC2 (3.7%)
PC1 (5.9%)
E
PCA based on promoters (GCH)
PC2 (6.2%)
PC1 (18.5%)
Sample
Control
HCM
F
PCA based on gene bodies (GCH)
PC2 (5.1%)
PC1 (24.9%)
Sample
Control
HCM
G
PCA based on enhancers (GCH)
PC2 (3.5%)
PC1 (6.6%)
Sample
Control
HCM

Fig S4

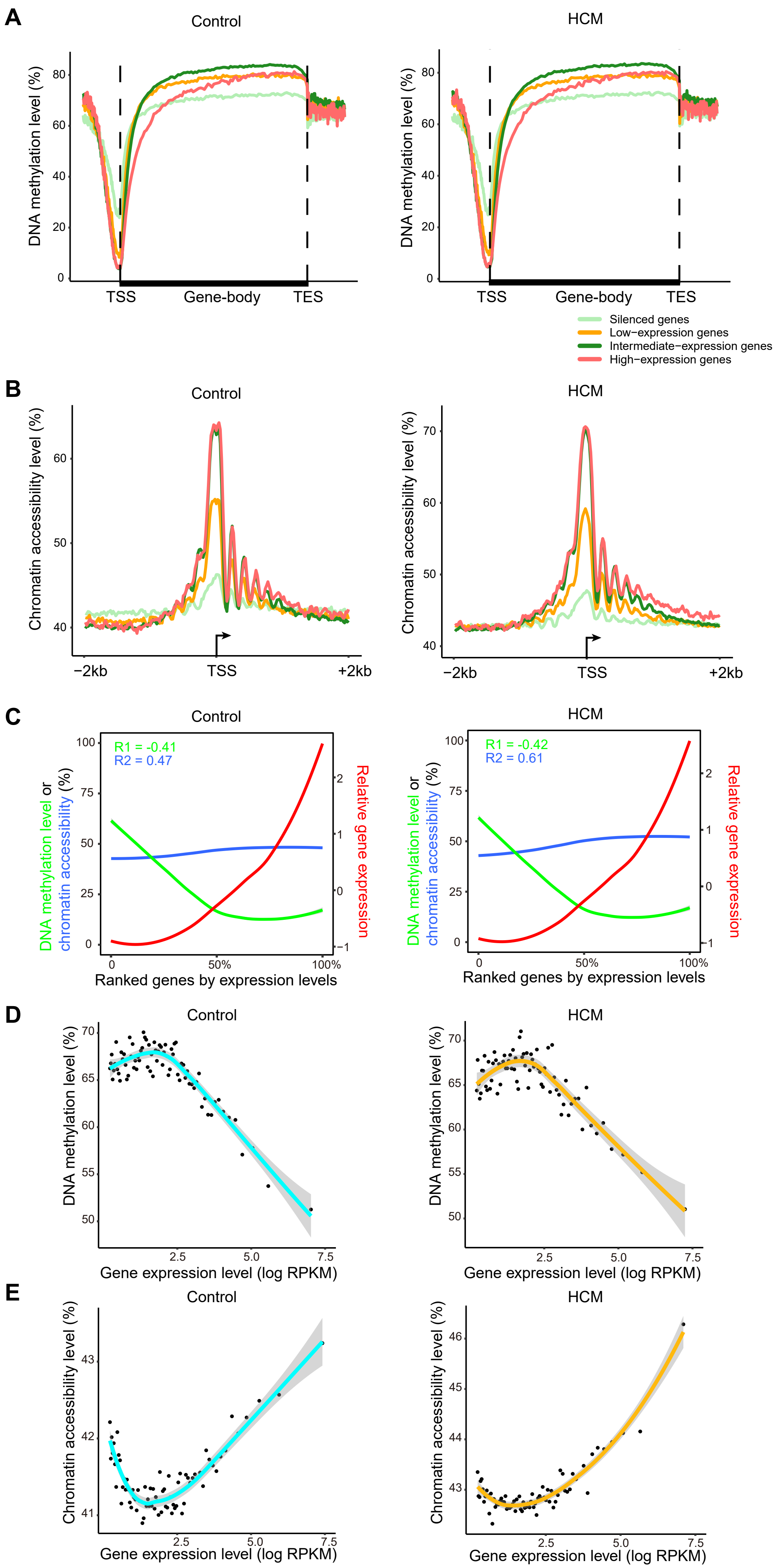
A
Control
HCM
DNA methylation level (%)
TSS
Gene-body
TES
Silenced genes
Low−expression genes
Intermediate−expression genes
High−expression genes
B
Chromatin accessibility level (%)
−2kb
+2kb
C
R1 = -0.41
R2 = 0.47
R1 = -0.42
R2 = 0.61
DNA methylation level or chromatin accessibility (%)
Relative gene expression
Ranked genes by expression levels
D
Gene expression level (log RPKM)
E
Chromatin accessibility level (%)

Fig S5

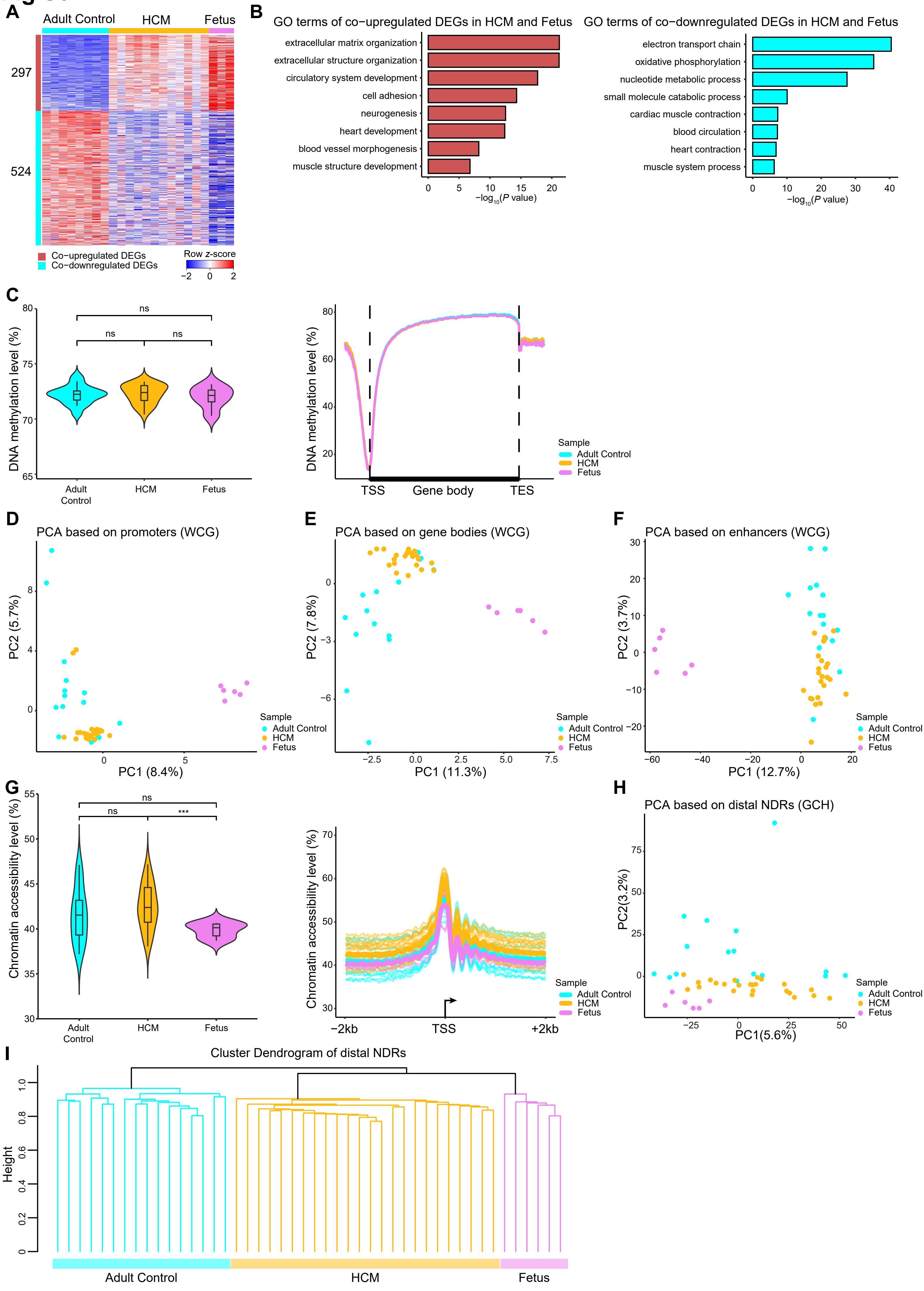
A
Adult Control
HCM
Fetus
297
524
Co−upregulated DEGs
Co−downregulated DEGs
Row z-score
−2
0
2
B
GO terms of co−upregulated DEGs in HCM and Fetus
extracellular matrix organization
extracellular structure organization
circulatory system development
cell adhesion
neurogenesis
heart development
blood vessel morphogenesis
muscle structure development
−log10(P value)
GO terms of co−downregulated DEGs in HCM and Fetus
electron transport chain
oxidative phosphorylation
nucleotide metabolic process
small molecule catabolic process
cardiac muscle contraction
blood circulation
heart contraction
muscle system process
−log10(P value)
C
DNA methylation level (%)
ns
Adult Control
HCM
Fetus
TSS
Gene body
TES
Sample
D
PCA based on promoters (WCG)
PC1 (8.4%)
PC2 (5.7%)
E
PCA based on gene bodies (WCG)
PC1 (11.3%)
PC2 (7.8%)
F
PCA based on enhancers (WCG)
PC1 (12.7%)
PC2 (3.7%)
G
Chromatin accessibility level (%)
***
−2kb
+2kb
H
PCA based on distal NDRs (GCH)
PC1(5.6%)
PC2(3.2%)
I
Cluster Dendrogram of distal NDRs
Height

Fig S6

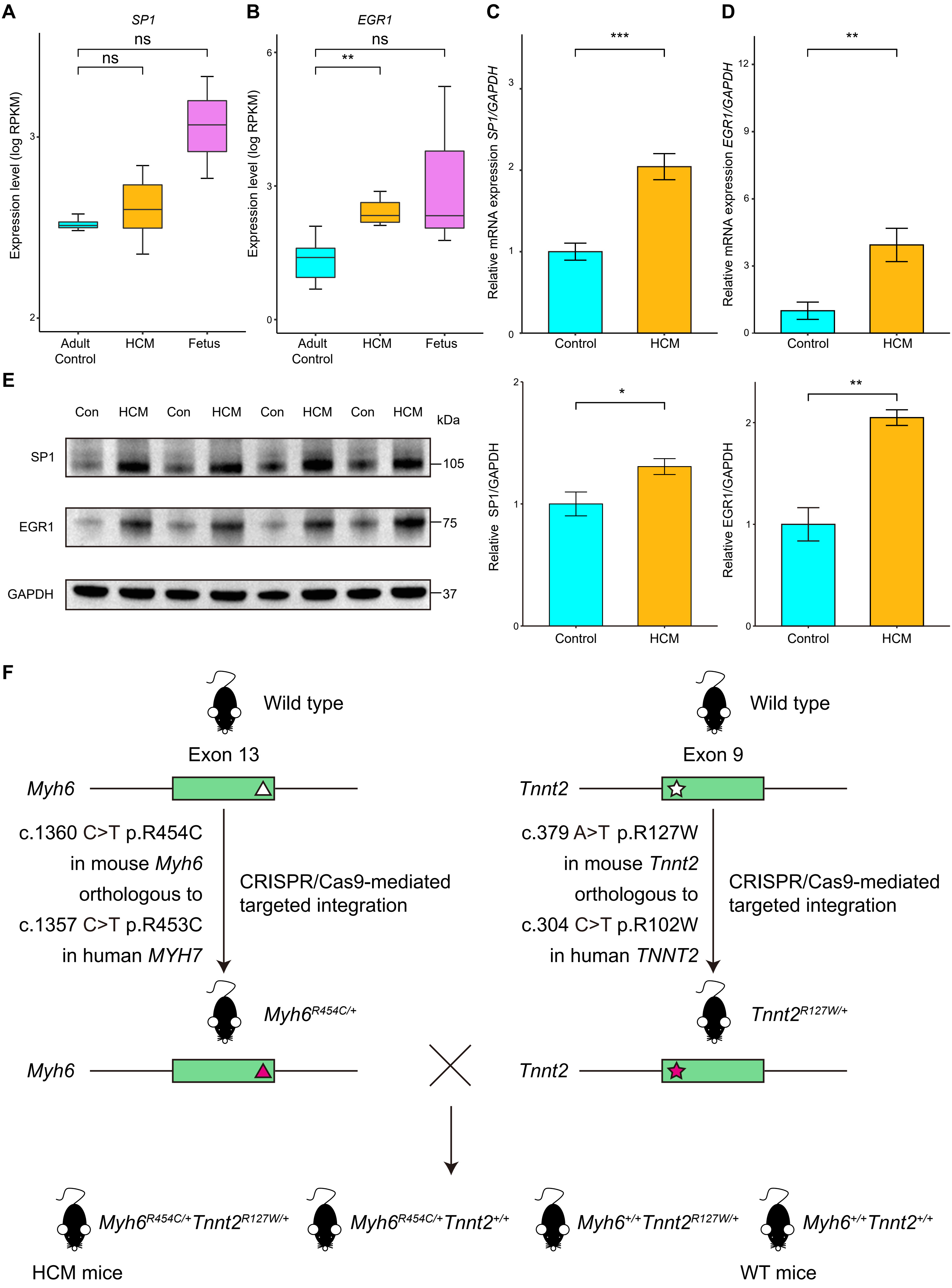

A
SP1
ns
ns
Expression level (log RPKM)
Adult Control
HCM
Fetus
B
EGR1
ns
**
Expression level (log RPKM)
Adult Control
HCM
Fetus
C
***
Relative mRNA expression SP1/GAPDH
Control
HCM
D
**
Relative mRNA expression EGR1/GAPDH
Control
HCM
E
Con
HCM
Con
HCM
Con
HCM
Con
HCM
kDa
SP1
105
EGR1
75
GAPDH
37
*
Relative SP1/GAPDH
Control
HCM
**
Relative EGR1/GAPDH
Control
HCM
F
Wild type
Exon 13
Myh6
c.1360 C>T p.R454C
in mouse Myh6
orthologous to
c.1357 C>T p.R453C
in human MYH7
CRISPR/Cas9-mediated
targeted integration
Myh6R454C/+
Myh6
Wild type
Exon 9
Tnnt2
c.379 A>T p.R127W
in mouse Tnnt2
orthologous to
c.304 C>T p.R102W
in human TNNT2
CRISPR/Cas9-mediated
targeted integration
Tnnt2R127W/+
Tnnt2
Myh6R454C/+Tnnt2R127W/+
Myh6R454C/+Tnnt2+/+
Myh6+/+Tnnt2R127W/+
Myh6+/+Tnnt2+/+
HCM mice
WT mice

Supplemental tables

Table S1. The clinical parameters of HCM patients.

Table S2. The sequence information of NOMe-seq and RNA-seq.

Table S3. DEGs (protein-coding genes and lncRNAs) between the adult controls and HCM patients.

Table S4. DMRs between the adult controls and HCM patients.

Table S5. Co-regulated DEGs in both fetuses and HCM patients compared with adult controls.

Table S6. Motifs enriched in distal and proximal NDRs in the adult controls, HCM patients and fetuses.

Table S7. DEGs between HCM mice and WT mice.

Table S8. DEGs between HCM mice and HCM mice treated with plicamycin or ML264.

Table S9. Co-regulated DEGs in fetal mice and HCM mice compared with WT mice.
